# Rapid Evidence Assessment of Mental Health Outcomes of Pandemics for Health Care Workers: Implications for the Covid-19 Pandemic

**DOI:** 10.3389/fpubh.2021.629236

**Published:** 2021-05-21

**Authors:** Sara Waring, Susan Giles

**Affiliations:** Department of Psychological Sciences, University of Liverpool, Liverpool, United Kingdom

**Keywords:** health care workers, pandemic, mental health outcomes, rapid evidence assessment, COVID-19

## Abstract

**Background:** Little is known about the long-term mental health (MH) impact of the Covid-19 pandemic on health care workers (HCWs). However, synthesizing knowledge from past pandemics can help to anticipate this, along with identifying interventions required, when, and target populations most in need. This paper provides a balanced evaluation of what is currently known about short- and long-term MH impacts of pandemics on HCWs and effect of methodological limitations on knowledge claims.

**Method:** A rapid evidence assessment (REA) was conducted on 41 studies published in the past two decades that examined MH outcomes for HCWs in relation to pandemics.

**Results:** Findings of literary synthesis highlight common MH outcomes across pandemics, including increased stress, distress, burnout, and anxiety in the short-term, and post-traumatic stress and depression in the long-term. Findings also show the key role that organizations and public health bodies play in promoting adaptive coping and reducing health worries and the emotional and psychological distress caused by this. Evidence highlights particular groups at risk of developing MH issues (contact with patients that are infected, having children), and time points where risk may increase (initial response phase, when quarantined). However, inconsistencies in measures, analysis, and reporting all create limitations for pooling data.

**Conclusions:** Findings can be used by researchers to provide a knowledge framework to inform future research that will assist HCWs in responding to pandemics, and by policy makers and service planners to provide an evidence-led brief about direction and evidence base for related policy initiatives, interventions or service programmes.

## Background

Previous pandemics have posed substantial risks to health care workers' (HCWs) physical (1–4) and mental health (5–8), even causing some to question career choices and affecting ability to treat patients (9–16). Whilst little is known about what the long-term mental health (MH) impacts of responding to Covid-19 will be, synthesizing knowledge from past pandemics that share similar features can help to anticipate this. Indeed, findings from a recent rapid evidence assessment (REA) and meta-analysis conducted by Kisely et al. (17) provide an informative overview of the short-term psychological effects of emerging virus outbreaks on HCWs. The review highlighted that HCWs were at risk of experiencing psychological distress, which was increased by exposure to patients with the disease, younger age, having dependent children, stigma, spending longer in quarantine, and limited support. The following paper seeks to build on this body of work, adopting an REA approach to focus specifically on pandemics and MH outcomes over time, from peak response and recovery, through to longer-term. The aim is to provide a critical and balanced assessment of what is currently known, methodological strengths and limitations, and recommendations for improving research quality within the fast-paced, dynamic context of pandemics. Findings will also pose important implications for identifying the form of interventions required for HCWs responding to Covid-19, target populations most in need, and at what points in time.

### Contextual Features

The past two decades have seen several viral outbreaks, including severe acute respiratory syndrome (SARS) in 2003, H1N1 influenza in 2009, Middle East respiratory syndrome (MERS) and Ebola in 2012, and now Covid-19. SARS, H1N1, and Covid-19 were classified as pandemics, with viruses spreading over multiple countries and impacting millions of people. Annually, viruses such as seasonal influenza result in 3–5 million cases of severe illness and a global mortality rate (GMR) of ~0.1% (18). Whilst the 2009 H1N1 outbreak was more infectious, resulting in 60.8 million cases in the US alone, the GMR was much lower at 0.001–0.007%. In contrast, the SARS outbreak was more deadly, with a GMR of 11%, but less infectious with 8,098 people worldwide becoming ill. The majority of infections were hospital acquired (4, 19) with HCWs comprising many cases [37–63% in the most affected countries (20–22)].

In comparison, 17 months after the initial outbreak of Covid-19 in Wuhan, more than 137 million cases and 2.95 million associated deaths were confirmed globally (23) (13th April 2021). The vast majority of fatalities were people over the age of 70 or with underlying health conditions. As with SARS, many HCWs are amongst these numbers. In March 2020, WHO initially estimated the GMR to be 3.4% (24) but the true figure is unknown given that symptoms are mild to moderate in 80% of cases and, until recently, many countries were predominantly only testing people with symptoms. What figures do indicate is that Covid-19 is more deadly than seasonal influenza and H1N1, and more infectious than SARS. Many who contract Covid-19 are asymptomatic or symptoms take several days to appear, which means they could be infecting others without knowing it. This combination of features poses substantial challenges for managing the pandemic and places health systems and HCWs under extreme burden.

### Pandemic Response

Pandemic response involves balancing ‘business as usual' to minimize economic impact with measures such as social distancing and quarantining to minimize health impact (25). Modeling studies show the value of these measures for delaying the overall virus impact to allow time for antiviral drugs to be administered and appropriate vaccines developed (26, 27). Both social distancing and quarantining were implemented during the SARS outbreak but were mainly restricted to people displaying symptoms or coming into contact with those displaying symptoms, which predominantly meant HCWs (28). Social distancing was implemented more widely during the highly infectious H1N1 outbreak in Australia and parts of America for short periods of a few weeks (29, 30). In contrast, Covid-19 has instigated the largest, most impactful global pandemic response seen in the past 100 years. Social distancing has been widely enforced in many countries for several months, along with travel restrictions. More stringent measures such as “lock down” have been mandated across general populations in an attempt to slow the virus spread and avoid healthcare systems from becoming overwhelmed.

In the UK, the response has also been unique in terms of composition of HCWs responding to the threat. More than 20,000 retired HCWs have returned to the NHS to provide vital support at a critical time. They are potentially facing increased risks as Covid-19 mortality rates are much higher in those over the age of 60 (31). In addition, thousands of final year medical and nursing students stepped in to provide frontline support. Whilst they possess important medical knowledge and skills, these students have limited experience of working in crises, particularly of a prolonged, complex scale. The HCW population responding to Covid-19 is therefore more diverse than would ordinarily be the case, ranging from experienced retired HCWs in an increased risk age bracket, through to very recently qualifying HCWs with limited experience. This raises questions regarding the MH impact of responding to Covid-19 for HCWs.

### Current Study

The current study provides a balanced assessment of what is currently known about the short- and long-term MH impact of pandemics on HCWs. We adopt a REA approach to address the need for timely evidence-based recommendations. Pooling data from across studies can identify links between key features and positive or negative MH outcomes, protective factors, and types of support needed, thereby identifying what interventions could benefit HCWs under the current pandemic and when. Such research is often conducted “as things happen” or retrospectively and so this REA will also assess evidence quality and implications for knowledge claims. This includes considering the extent to which data may be pooled given cross-cultural variation in a range of issues. Review findings can be used by (a) researchers to provide a knowledge framework of issues that have emerged in past research and inform development of data proformas to improve comparability of evidence in future research; and (b) policy makers and service planners to provide an evidence-led brief about direction and evidence base for policy initiatives, clinical interventions or service programmes.

## Method

An extensive search was conducted for studies published in peer-reviewed journals between January 2000 and May 2020 in order to include evidence emerging from the SARS outbreak in 2003. As the MH outcome of pandemics on HCWs is a multidisciplinary topic, the following databases were used: Cochrane Library, Discover, PsychInfo, PubMed, Science Direct, Scopus, and Google Scholar. In line with Varker et al.'s (31) general principles for conducting REA, we consulted with knowledge users from clinical and health settings to determine the scope of the research question, purpose, and search, inclusion and exclusion criteria (Table 1). The search strategy sequence was: (1) mental health outcomes^*^ OR mental OR psychological OR health OR emotional OR social OR costs OR consequences; (2) health care workers^*^ OR healthcare workers OR medical workers OR doctors OR physicians OR nurses OR paramedics; (3) pandemic^*^ OR epidemic OR protracted incident.

**Table 1:** Inclusion and exclusion criteria used for the rapid evidence assessment.

**Population**	**Health care workers responding to pandemics, epidemics or protracted incidents**
Inclusion criteria	- Papers (peer reviewed academic journals) focused on health care workers in high and upper-middle income countries - Paper written in English - Only papers where the full-text version is readily available - Includes empirical data on a) mental health outcomes (e.g., observational/ prevalence) or b) economic costs attached to mental health outcomes
Exclusion criteria	- Focus on health care workers in low and lower-middle income counties - Not available in English - Full-text version is not readily available - No empirical data on mental health outcomes or economic modeling of mental health outcomes

**Figure 1:**
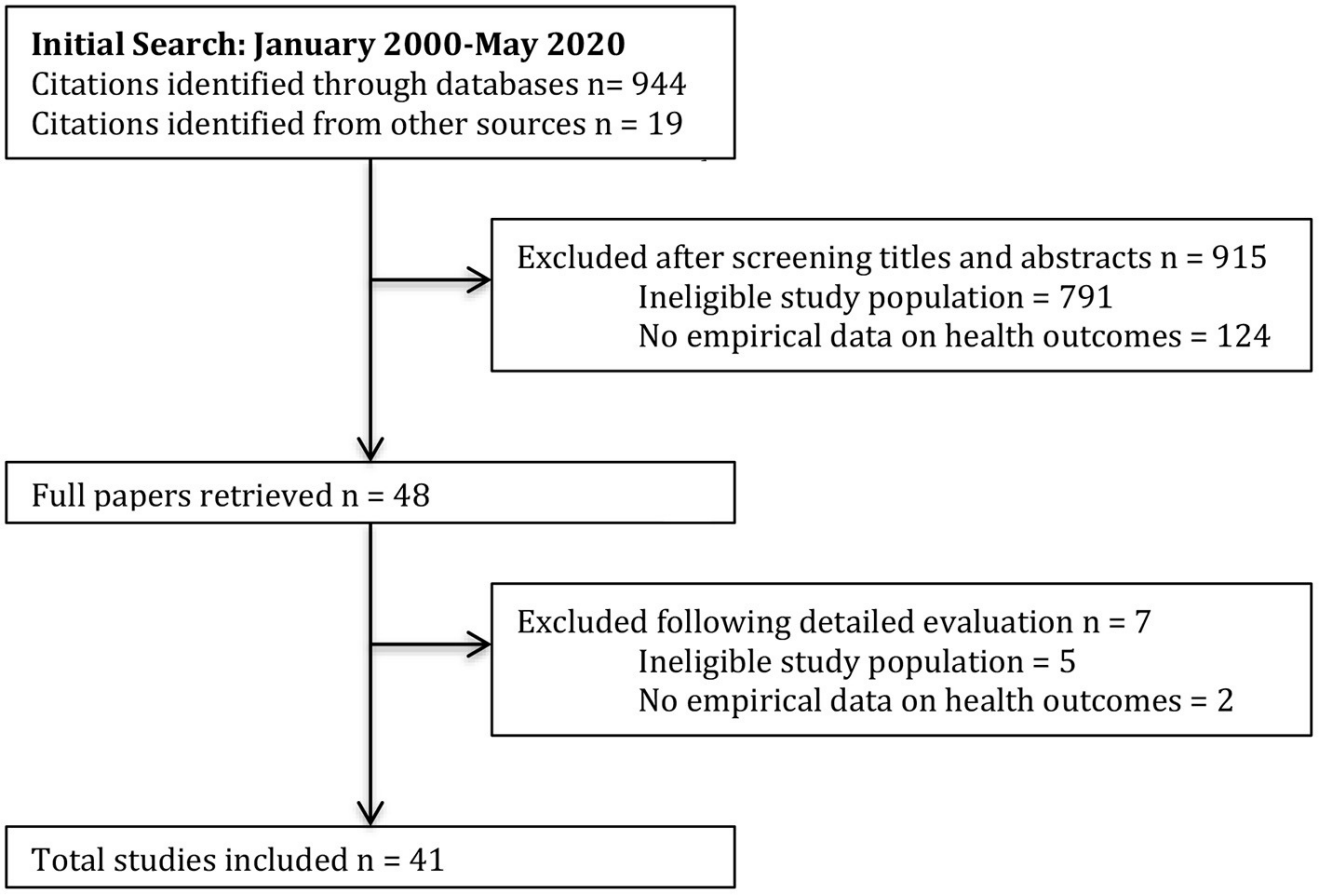
Flow diagram of study selection adapted from “Preferred items for systematic reviews and meta-analyses: the PRISMA statement,” by Moher et al. (32), PLoS Medicine, 6(7).

Data extraction followed a two-stage selection process proposed by Varker et al. (31) to improve method transparency and standard of quality assessment, bringing REA more in line with guidelines (such as NICE) for providing summaries of the strengths and weaknesses of studies, applicability issues and other relevant contextual points. The first author carried out the initial screening of titles and abstracts against the predetermined inclusion/exclusion criteria. Where the relevance of the article was unclear, the full-text version was downloaded. The same reviewer read the full-text version and decided whether the paper should be included or excluded based on the pre-defined criteria (see Figure 1). Inter-rater reliability was conducted with another reviewer independently reading 50% of articles to assess whether they met the inclusion criteria, resulting in 95% agreement. Differences of opinion between the reviewers were resolved through discussion, resulting in 100% agreement. In total, 41 studies were identified as relevant to include in the REA.

A data extraction form was developed based on PRISMA guidelines (33). Studies were selected and appraised in respect of the Population, Interest, Comparisons and Outcomes (PICO) framework (34). Study quality was assessed using the STROBE checklist (35) and FORM framework (36) adapted by Varker et al. (37) for REA. Quality assessment was undertaken by the second author in consultation with the first author.

## Results

The majority of the 41 studies adopted a quantitative cross-sectional survey design to measure mental health outcomes but a range of different validated measures were used (see Table 2 for details of study composition). In addition, 29 studies used questionnaires designed by authors to measure issues such as exposure to infected patients, coping strategies, training adequacy, perception of support, impacts on health, personal relationships and work life, disease knowledge, and risk acceptance. Details of how these scales were developed, the specific constructs they measured, or evidence of validation were often limited. Sampling frames ranged from all staff in one hospital to staff from across 34 hospitals. Some studies focused on doctors or nurses only whilst others included a variety of professionals such as paramedics, healthcare attendants, health administrators, pharmacists, physiotherapists, and psychiatrists. Traditional Chinese medicine practitioners, cleaners, food service providers, workmen and transport workers were also included in single studies. Only 13 studies involved an aspect of case-control and the nature of control groups varied from general population (*k* = 2) and traditional Chinese medicine practitioners (*k* = 1), to HCW's in lower risk work environments (*k* = 8), involuntarily conscripted to work in high-risk units (*k* = 1) and recovering from the virus (*k* = 1). Typical experience ranged from >2 to <33 years but many studies did not report details of experience, gender, age, or number of participants in each role.

**Table 2:** Composition of study population and data collection methods and measures used.

Incident	- 2003 SARS outbreak (*k =* 34) - 2009 H1N1 outbreak (*k =* 4) - Covid-19 (*k =* 3)
Country	- Canada (*k =* 11) - Asia (*k =* 27; Hong Kong = 8, Taiwan = 8, Singapore = 5, China = 5, Japan = 1) - Australia (*k =* 2) - Greece (*k =* 1)
Methods and measures	Quantitative (*k* = 36) - Psychiatric morbidity ∘ General Health Questionnaire (GHQ 12 and 28; *k =* 8) ∘ Chinese Health Questionnaire (CHQ; *k =* 3) ∘ Symptom checklist-90 (*k =* 2), Center for epidemiologic studies depression scale (*k =* 2) ∘ Zung's self-rating Anxiety Scale (*k =* 2) ∘ Patient Health Questionnaire (PHQ; *k =* 1) ∘ Generalized Anxiety Disorder Scale (GAD; *k =* 1) ∘ Depression and Anxiety Scale (DAS; *k =* 1) ∘ Beck depression inventory (*k =* 1), Zung's self-rating depression scale (*k =* 1) ∘ Spellberg Trait Anxiety Inventory (*k =* 1) ∘ Diagnostic Statistical Manual (DSM) clinical guidance (*k =* 1) - Stress ∘ Perceived stress scale (*k =* 2) ∘ Kessler psychological distress scale (*k =* 2) ∘ State trait anger expression inventory (*k =* 3) - Post-traumatic stress symptoms ∘ Impact of Events Scale (IES & IES-R; *k =* 18) ∘ Davidson trauma scale-Chinese (*k =* 2) ∘ Clinically administered PTSD scale (*k =* 1) - Coping strategies ∘ Coping Orientations to Problems Scale (COPE; *k =* 2) ∘ Chinese self-efficacy scale (*k =* 1). - Physical aspects (such as burnout, insomnia and disabilities) ∘ Maslach burnout inventory (k = 5) ∘ m Pittsburgh sleep quality index (*k =* 2) ∘ Insomnia severity index (*k =* 1) ∘ Sheehan's disability scale (*k =* 1). - Family background ∘ Parental bonding instrument (*k =* 2) ∘ Family APAGAR Index (*k =* 1) - Personality Eysenck's personality inventory (*k =* 2). - Eisenberger et al. Survey of perceived organizational support (*k =* 1) Qualitative (*k* = 5) - Combination of open-ended questionnaires, interviews, focus groups, and observations (*k =* 4) - Interviews only (*k =* 1)

Although we initially intended to conduct a meta-analysis on the data extracted from the 41 papers, this was not possible due to the wide variety of differences in measures used, mental health outcomes assessed, statistics used, populations, case controls, and time points over which data was collected (38). For example, the GHQ was the most commonly used measure (*k* = 8). Three of these studies were conducted in Singapore 2 months after the initial SARS outbreak, but with different samples (nurses and doctors vs. physiotherapists, occupational therapists and speech therapists vs. general practitioners and traditional Chinese medicine practitioners). One study was conducted in Canada at the peak of the SARS outbreak with healthcare professionals, nurses and doctors. Another was conducted in Hong Kong with nurses and healthcare assistants during the beginning of the post-SARS recovery period. One was conducted in Singapore 6 months post SARS recovery with nurses and doctors. Another study relating to H1N1 was conducted mid-outbreak in Greece with nurses, medical, allied health and auxiliary staff. A study relating to Covid-19 was conducted during the peak of the initial outbreak in China with nurses and doctors. All other measures were used in three or less studies, each of which examined different populations across different time periods. We have therefore conducted a narrative synthesis, using an interpretive approach to extrapolate meaning and understanding across studies (39, 40).

In the following section, findings are structured in relation to the following five recurring key themes that were present across studies: (i) timing of research; (ii) contact with patients; (iii) age and years of experience; (iv) gender; and (v) communication and confidence in training and equipment.

### Timing of the Research

Studies varied in terms of whether they focused on short (*k* = 31) or longer-term (*k* = 10) MH outcomes. Short-term chronology of studies can be broken down into data collected during the initial phase of outbreak (i.e., first few weeks; *k* = 1), peak infection (*k* = 14), and recovery phase (i.e., no new cases recorded, or services start to return to normal; *k* = 13). Three studies did not provide details of when data was collected other than that it was during the outbreak. The 10 studies that focused on longer-term outcomes were conducted six (41, 42), eight (43), 12 (43, 44), 13–26 (45), 16–27 (46), 24 (47), 30 (48), and 36 months (49) after services began returning to normal. Only five studies examined outcomes at different time points, comparing initial with repair phases (5, 50), repair phase with 8 months later (43), and infection peak (44) or recovery phase (51) with 12 months later. Three studies drew samples from the same hospitals but not necessarily the same participants. Two studies used the same participants and made direct comparisons (50, 51).

### Short-Term

Studies highlight associations between pandemics and increased stress (5, 6, 44, 52–57), emotional and psychological distress (5, 10, 33, 54, 57–62), burnout and exhaustion (60), anxiety (5, 9, 54, 56, 60, 61), post-traumatic stress and depressive affect (5, 54, 57, 61, 63–66). The majority of these studies were conducted in relation to the SARS outbreak but similar outcomes were identified across a range of countries, including Australia, Canada, China, Hong Kong, Singapore and Taiwan. Distress, and anxiety were also measured in relation to the H1N1 outbreak in Australia, Greece and Japan. Three studies published in relation to Covid-19 also highlight similar MH outcomes in China, including anxiety, distress, depression and insomnia (8, 54, 67).

In relation to stress, findings showed that HCWs experienced similar levels to members of the public during the SARS outbreak, but this was still much higher than for other significant life events such as unemployment and separation (53). HCWs attributed this elevated stress to the pandemic (52, 55), and were more likely to experience increased stress if they were married with children at home (6) or conscripted to work in a high-risk ward rather than volunteering (68). Those who were conscripted also reported more symptoms of intrusion, depression and psychoticism (68). HCWs families were also experiencing stress and roles and routines were affected, including partners taking on additional responsibilities such as shopping, school drop off and childcare (56). In one study, 74.2% of HCWs reported this increased stress was still negatively affecting family relationships as the pandemic moved into the repair phase (5).

Many HCWs also reported experiencing negative effects such as tiredness, health worries and fear of social contact (40, 52, 53, 63, 69, 70). Health worries about contracting and passing on SARS and H1N1 to family were repeatedly associated with stress, distress, anxiety and burnout (5, 6, 10, 33, 44, 53, 56, 59–61, 71, 72). Health worries were greater if HCWs had children at home (6, 10, 33, 56, 58), perceived themselves to be at increased risk of contracting the virus and becoming seriously ill (6, 25, 33, 44, 56, 57, 59, 60, 72), and knew or cared for fellow HCWs that had contracted the virus (56, 71). Studies also highlight that HCWs felt stigmatized by friends and neighbors and believed their family were stigmatized as a result of the job they did (6, 10, 25, 40, 56, 61, 71). Other substantial intrusive life impacts included staying away from home to minimize risk of infection (56, 60), changes in teaching, research, and ability to deliver patient treatment (10, 25, 60). For HCWs still living away from families during the repair phase, emotional distress was significantly more likely (5), with family support being identified as a protective factor against developing anxiety and depression (50). For HCWs who were quarantined, feeling socially isolated was also common (56), and was associated with increased psychological distress (63).

Findings from one study comparing outcomes across short-term phases showed that stress, anxiety, and worries about contracting and passing SARS on to family were higher during the initial response, whereas depression, post-traumatic stress and somatic symptoms were more likely during the recovery phase, as was considering resigning (5). One study conducted in Taiwan demonstrated the value of organizational interventions that focus on providing infection management training, detailed manpower allocation, adequate personal protective equipment (PPE) and access to MH teams for reducing anxiety and depression and improving sleep quality for nurses treating patients with SARS, both during peak response and recovery phases (50).

### Long-Term

All longer-term studies focused on the SARS outbreak. Findings highlighted similar MH outcomes to short-term studies, including elevated stress (44, 51), psychological distress (45, 54, 73), burnout (41, 45), anxiety (44), state anger (41), post-traumatic stress and depression (42–44, 48, 49). Having a friend or relative that contracted the disease, being single, and having a low household income were associated with higher post-traumatic stress symptoms over two and a half years after the outbreak (48). Persisting health worries more than 6 months after the outbreak were associated with increased emotional exhaustion and state anger (41). Adaptive coping was identified as a protective factor against experiencing long-term post-traumatic stress, burnout and psychological distress (45). However, spending longer periods socially isolated in quarantine hindered adaptive coping (73), and increased risk of developing post-traumatic stress and depressive symptoms (40, 42, 48, 49) up to 3 years post-outbreak.

In the single study that compared short- and longer-term outcomes, stress levels were higher a year post-outbreak in HCWs at high risk of exposure to SARS but dropped in HCWs less likely to come into contact with infected patients (44). The authors suggest this may be the result of frustration at not receiving recognition for their contribution to the response, or anticipation of new virus outbreaks. Longer-term studies also focused on issues relating to productivity and secondary health problems, highlighting multiple adverse outcomes, including increased absence, reduced ability to maintain patient contact, and substance abuse (45).

### Contact With Patients

A number of studies highlighted that working in departments at greater risk of coming into contact with and treating infected patients increased likelihood of developing a variety of difficulties compared with HCWs at low risk of coming into contact with infected patients. These high risk HCWs were significantly more likely to be concerned about contracting and spreading SARS to family members (6, 10, 41, 52, 67), which appears to be justified given that they were also more likely to be quarantined (25, 41). High risk HCWs were more likely to have changed living arrangements to minimize risks to family (10) and to experience greater difficulties in getting along with friends and family (55). They also reported feeling significantly more stigmatized (10), significantly greater negative responses, including fatigue, poor sleep, health worries, and fear of social contact (44, 66). Whilst high-risk HCWs noted feeling greater camaraderie with staff working and facing the situation together, some conflict was reported toward “non-essential” workers who remained at home and were paid (56).

High risk HCWs were also more likely to develop a range of MH problems. In the short-term, this included elevated stress (6, 57), distress (5, 10, 61), anxiety (5, 60, 61, 72), burnout and exhaustion (20, 41, 60, 73), state anger (41), post-traumatic stress (5, 20, 55, 63, 66), and depression (5). Being a nurse was linked with increased stress and distress in the short-term (33, 54, 60, 62) and post-traumatic stress symptoms 6 months post- outbreak (42) but this may relate to nurses having more direct contact with patients. Similar findings were recently observed in frontline HCWs responding to Covid-19 in Wuhan, the most affected region of China, who reported more severe depression, anxiety, distress and insomnia (54).

The level of contact HCWs had with infected patients continued to impact MH long-term, with stress (44), burnout, psychological distress, posttraumatic stress and depression (63) being significantly higher a year after the SARS outbreak for HCWs that were at high-risk of contact. Productivity was also more severely affected over a year post-pandemic including reduced patient contact and work hours, and increased substance use and days off work (45). Indeed, high risk HCWs were twice as likely to experience multiple problems than HCWs at low risk of coming into contact with infected patients (45). Whilst most of these studies were conducted in relation to the SARS outbreak, findings from across a number of countries demonstrate similar findings.

### Age and Years of Experience

Seven studies highlighted the role of age or years of experience as a risk factor for MH difficulties. Four related to the 2003 SARS outbreak in China (49), Taiwan (5), and Hong Kong (57, 74), two to the 2009 H1N1 outbreak in Australia (5) and Japan (20), and one to the Covid-19 outbreak in China (72). In China, depressive symptoms were higher in younger HCWs 3 years post SARS outbreak (49). In Hong Kong, younger HCWs reported greater job-related stress (57) and negative impact on quality of life (74) during the SARS outbreak. In Taiwan, short-term distress was greatest in those with < 2 years experience (5). In Japan and Australia, younger HCWs experienced significantly greater anxiety about contracting H1N1 (20, 58), causing some to refuse to care for patients (58). Recent findings in relation to Covid-19 also highlight increased anxiety and hostility in younger HCWs (72). However, a further six studies found no age differences in anxiety (8), distress (62), psychiatric morbidity (43, 51, 55), post-traumatic stress or depressive sympotoms (64, 75). A further study also report that experience had no significant impact on post-traumatic stress (6). Overall, study numbers are limited, each one focuses on different MH constructs, and a variety of measures are used, making it difficult to draw firm conclusions but there are some indications that having less healthcare experience may increase risk of short-term stress, distress, anxiety and long-term depression.

### Gender

Six studies highlight gender differences in MH outcomes. In relation to the SARS pandemic, men reported experiencing greater emotional distress in the short-term in Taiwan (5) and short- and long-term stress in Hong Kong (44). In contrast, women were at increased risk of experiencing more severe symptoms of depression and anxiety in the short-term in relation to the SARS outbreak in Hong Kong (57), Taiwan (5), and the on-going Covid-19 response in China (54). Women were also significantly more worried about infecting family during the SARS outbreak (74). However, a further seven studies reported no gender differences in distress (10, 62), anxiety (8), psychiatric morbidity (51) or post-traumatic distress (6, 43, 55). Findings of these few studies are mixed but there are some indications that men may be more likely to experience stress and emotional distress, whereas women may be more likely to experience health worries, anxiety and depression. However, all of these studies focus on HCWs in Asian countries. Little is known about whether gender differences exist in western countries.

### Communication and Confidence in Training and Equipment

During the highly infectious 2009 H1N1 outbreak, the supply of essential equipment and consumables was raised as a concern for health systems (9). As PPE supplies ran low, guidelines of what was considered appropriate and sufficient PPE changed (58). These inconsistencies in PPE protocol and issues with availability were associated with lower trust in protective measures and increased health worries about infection, both in relation to H1N1 (58) and SARS (10, 76). Lower trust in protective measures was also associated with increased stress (53, 76) and anxiety (60) in the short-term, and burnout, psychological distress, post-traumatic stress (45), state anger, and avoidant coping (73) in the long-term. Trust in protective measures appears to play an important role in short- and long-term MH outcomes for HCWs.

Findings also highlighted that public health bodies and HCWs' organizations played an important role in promoting trust in protective measures. Level of perceived access to transparent, trustworthy information regarding virus prognosis (59), protective measures and rationale for changing these measures (60), affected HCWs' levels of trust, stress and health worries. HCWs reported feeling angry about the spread of SARS and lack of or conflicting information given by management and public health bodies (56, 75). They reported experiencing stress and health worries as a result of seeing their children frightened and finding it difficult, in the absence of adequate support from public health bodies, to explain the situation without causing more fear, or to be confident about minimizing infection risks at home (56). There was also frustration that the spread of SARS could have been curtailed if HCW concerns had been heard and vigilant safety precautions quickly implemented (56). Perceived lack of organizational support continued to impact HCWs 6 months after recovery, leading to increased exhaustion and state anger (41). Providing adequate training and support served as protective factors against developing state anger (73), avoidant coping (45, 73), depression and post-traumatic stress (57, 77).

## Discussion

The purpose of this RAE was to provide a balanced assessment of what is currently known about the short- and long-term MH impact of pandemics on HCWs. Most studies included are best described as being discovery focused, conducted in naturalistic settings and drawing on unselected samples of HCW populations. The variation across studies precluded meaningful data pooling; rather this paper has provided a synthesis of meaningful themes that can guide decision making in the current pandemic. Applicability is promising as, despite evidence emerging from three different pandemics over a 17-year period, there is consistency in findings across a range of countries, including Australia, Canada, China, Greece, Hong Kong, Japan, Singapore, and Taiwan. Below, we discuss the findings of the REA, highlighting consistencies in the direction of results across studies. This is followed by a discussion of quality assessment, and finally implications and recommendations.

### Mental Health Outcomes

Findings of this REA parallel a recent meta-analysis conducted by Kisely et al. (17), indicating that during peak pandemic response, HCWs experience increased stress, distress, anxiety and burnout. In addition, the current REA also focused on longer-term outcomes as services return to normal functioning with findings highlighting additional MH problems, including post-traumatic stress and depression. Several studies showed that risk of developing short- and long-term MH problems is associated with increased health worries, particularly if HCWs know or treat colleagues that are infected, have lower trust in protective measures and feel the information, training and support provided by public health bodies and their organization is inadequate. Whilst adaptive coping, social and family support serve as protective factors against developing MH problems, these are compromised when HCWs spend longer periods in quarantine. With over 850 UK HCWs losing their lives to COVID-19 to date, and concerns also repeatedly being raised about PPE, changing protective advice issued by public health bodies and adequacy of protective measure, it is likely that HCW health worries in relation to COVID-19 have been exacerbated.

Findings from across a number of countries also consistently demonstrate that working in departments that are likely to come into contact with patients infected with the virus increases risk of developing a range of short- and long-term MH difficulties. Similar findings are beginning to emerge in relation to Covid-19 in China (8, 54, 72), including three studies that have not been included in this RAE as they are under review for publication (3, 47, 67). A small number of studies also highlight gender differences in MH outcomes, with men more likely to experience stress and distress and women more likely to experience post-traumatic stress and depression. A small number of studies additionally show that less experienced HCWs are more likely to report distress. These findings should be viewed with caution due to differences in how constructs were operationalized and a small number of other studies reporting no significant gender or age differences. However, they do pose some tentative implications for the UK Covid-19 response as a large number of recently qualified HCWs are working on the frontline.

Overall, in reviewing the relevance of these broad findings to COVID-19, it is important to consider pandemic context. For example, Chong et al. (5) found a large proportion of HCWs were unwilling to risk caring for patients with SARS in the initial phase and considered resigning in the repair phase due to continued fear of infection. Compared to Covid-19, SARS was less infectious but had a higher GMR and the majority of HCWs in Chong et al.'s study believed they would have little chance of survival if infected. Researchers examining the impact of Covid-19 may not replicate findings about unwillingness to work, partly because of lower GMRs but also due to societal-wide government lockdown measures (to reduce rate of infection) and decisions to keep children of key workers in school settings (helping to alleviate practical concerns raised by HCWs in papers reviewed). The large-scale national and international responses may mean some early intervention points have been addressed. Notwithstanding this, similarities in pressure on health care persist (respiratory equipment, PPE, need to ensure safe working practices), which indicate that similar MH outcomes are likely to emerge from Covid-19.

### Quality Assessment

The majority of studies used descriptive cross-sectional surveys, which is understandable given the unforeseen, often intense, risky and frenetic working conditions of HCWs during pandemics. Despite this, sample sizes were commendable, ranging from 47 to 10,511 for quantitative studies. Power and sample size calculations were not routinely undertaken but, with the exception of a few small-scale studies, the evidence base does not appear to suffer from underpowered studies. However, despite some authors recognizing the need for a high response rate so as to avoid under or overestimating the prevalence of psychiatric morbidity, internal validity was compromised by inability to make comparisons between respondents and non-respondents due to the need to maintain anonymity [one study provides a representativeness survey (45)]. Measurement of relationships between communication and confidence in protective measures and MH outcomes are subject to similar issues as it is not possible to know whether HCWs with poorer MH states or lower trust in protective measures were more likely to participate. Studies would benefit from a clearer understanding of baseline psychiatric morbidity measures for comparing pre- and post-pandemic measures. Whilst cohort studies are understandably challenging, other existing surveys could provide meaningful aggregate baselines (7). Explicit focus on pre-existing MH amongst respondents is needed to improve internal validity of findings. Whilst this might compromise anonymity, research collaborations with blind researchers and aggregate reporting methods could help to circumvent this problem.

Another issue that compromises this body of evidence is variations in study design, providing little in the way of systematic replication, nor standard statistical comparisons between subgroups of participants. Too many studies provide bespoke measures (sometimes where validated scales exist), and too little information is provided about how constructs were defined or operationalized. Sampling frames also vary considerably, and too little information is provided about sample characteristics in general. It would be difficult to replicate the sampling frame or study materials of some studies without consultation with authors. The timing of research further compromises comparison; MH outcomes are collected at different time points and the exact timing of research is not clear from a small number of studies. Overall, the most compelling evidence relates to high risk HCWs and, even here, definitional variation exists with some studies defining high risk as treating patients that are infected or working in high dependency units where there are likely to be patients that are infected, through to working in hospitals where there were cases of the disease.

It is also important to note the limitations of this REA. Despite conducting a broad search, we were unable to locate the full text in English for five studies that might have been relevant. Three only published the abstracts in English, and the other two did not have full texts available. In addition, three papers were also identified that provided evidence of the MH outcomes for HCWs in China responding to Covid-19 (8, 46, 72), which are likely to provide useful comparisons against the outcomes of previous pandemics. Yet they were not included in the current REA because the papers have not yet been reviewed and published, and so may be subject to further analysis and reporting changes in the final published versions. The REA also only identified a single paper that focused on a prevention programme that was implemented in Taiwan during the SARS pandemic, which demonstrated improved anxiety, depression, and sleep quality scores (50). Consequently, conclusions drawn about efficacy of pre-existing interventions that have been used during previous pandemics to improve MH outcomes for HCWs are limited.

### Implications and Recommendations

Findings of this RAE provide a knowledge framework that can be used by researchers to inform future studies to assist HCWs in responding to pandemics. Observational studies are a vital part of responding to critical incidents and bio-disasters as they occur. Yet, the quality of observational studies could be much improved. The first recommendation is that authors make use of STROBE guidelines (22) to design their study. This would result in better descriptions of participants, thereby improving external validity; and methods thereby addressing problems with consistency and replicability. Questionnaires are particularly useful when lockdown procedures are in place. However, researchers are advised to use validated instruments where possible and be mindful of cross-validation with other instruments where it exists; for example, using GHQ (and measures with established concurrent validity such as CHQ, PHQ, GAD, and DAS). The IES-R might also be used (rather than IES) ideally 1 month after the initial phase so as to meet American Psychological Association guidelines around post-traumatic stress. Where cross-validation studies do not exist, it would be useful for researchers to undertake this work.

Whilst pandemics are unforeseen and uncontrollable, evidence from this review points toward similarities in constructs and research questions posed during three separate pandemics over a 17-year period, each involving respiratory disease. There have been similarities in concerns around PPE, quarantine and organizational communication that would have lent themselves to developing standardized and validated questionnaires. In reality, bespoke measures were used, and some authors provided too little information to aid replication. It would be useful for national research centers to work proactively to get ahead of this for the next potential pandemic. We recommend that national and international organizations (Chief Medical Officers, Centers for Public Health, WHO, CDC) develop a minimum data standard (or question bank) to capture organizational aspects and short- and long-term MH outcomes. This should include standardized measures to identify HCWs as “high-risk,” guidance on population descriptors and how they might be applied internationally, response bias, suitable controls and concurrent validity. Relatedly, researchers need to consider the timing of research. A sensible framework suggested from the findings of the current review is the tripartite structure, initial phase of outbreak, peak of infection and recovery phase. Follow up time periods might then also become more standardized. Having national and international data standards and question banks would help researchers to conduct an observational study that is more directly comparable with that of others, thereby helping to improve the quality of the evidence base. A potential question bank might also be collated for other diseases, or at least be open enough to be useful, if the next pandemic is a non-respiratory disease.

The review also set out to identify target groups for treatment and intervention points and findings will benefit treatment providers as well as hospital managers and those in strategic roles. The first finding is that HCWs that are exposed to infected patients are more likely to experience short- and long-term MH outcomes and should be a treatment priority. Following from this, there are three key sub-groups. HCWs with children in the home may benefit from targeted psychological support to help them to cope with worries about infectivity. Longer periods of quarantine appeared to hinder adaptive coping for some HCWs and may be a particular sub-group in need of support to promote adaptive coping and minimize risks to MH. The third key sub-group may be those with existing MH problems. Existing MH was conspicuously absent from the majority of studies and evidence that did exist pointed toward HCWs with existing MH problems experiencing stress in the short term. These individuals may need additional support during the pandemic peak followed by close long-term monitoring.

The limited evidence emerging from this review around uptake and feedback on interventions somewhat challenges the view that high-risk HCWs require psychological intervention in the short-term. Uptake of counseling services were low in relation to SARS (57) and HCWs responding to Covid-19 in Wuhan (54, 78) have argued that psychological interventions competed with much needed rest breaks. A number of studies point toward a particular camaraderie amongst high-risk HCWs in a crisis; a psychological buffer that protects mental well-being as well as perhaps explaining the poor uptake of services. HCWs in Wuhan identified a number of practical issues they did need assistance with, such as training on how to respond to patients and visitors that did not want to follow quarantine procedures. This has led academics and researchers to respond directly to these requests (e.g., https://www.liverpool.ac.uk/project-ares/communication/). Whilst it is beyond the scope of this review to directly challenge HCWs' views, nor of MH professionals wishing to provide short-term interventions, lessons emerging from this review point toward the need for practical solutions (such as help with childcare). MH input should be voluntary, easily accessible and designed with HCWs' work schedules in mind (4). For example, voluntary short courses on resilience and adaptive coping could help to minimize risk of experiencing long-term post-traumatic stress, burnout and psychological distress for some HCWs.

Further key findings point toward the role of organizations in promoting adaptive coping in the short term. Findings indicate the importance of communication and organizational response during these early stages to improve short- and long-term MH outcomes. Of further use to hospital managers and those in strategic roles are resource implications. Findings highlight that capacity to respond is not only likely to be affected in the short-term (e.g., for those with flu like symptoms and in quarantine, those experiencing emotional exhaustion). Once the pandemic moves into the recovery phase there are likely to be additional adverse effects (reduced productivity and performance, sick leave related to PTSD and emotional exhaustion). These findings indicate a need for public health bodies to take steps to address shortfalls in staffing and productivity that are likely to continue months and years beyond an outbreak. A programme of psychological support for high-risk HCW workers would be beneficial during the repair phase. High-risk HCWs also report increased secondary health problems (including increased substance use) and at this point health psychology interventions might be proffered on a national basis. The lack of long-term follow up and variation in follow up period limits firm conclusions about the timing of such adverse effects but hospital managers should expect resource problems to occur 12 months post-pandemic. High-risk HCWs should be able to draw on voluntary sources of psychological support for at least 12 months following the peak of a pandemic, ideally longer for particularly high-risk HCWs.

## Conclusion

This REA set out to synthesize knowledge from past pandemics to shed light on the potential impact of Covid-19 on HCWs. Forty-one studies were reviewed, the majority of which were discovery focused, conducted in naturalistic settings and drawing on unselected samples of HCW's populations. Although the variety in study designs, populations, measures and time periods precluded meaningful data pooling, we provided a narrative synthesis of themes that can guide decision making in the current pandemic. Target populations for intervention include high-risk HCWs with additional support needed for quarantined HCWs and those with children. Preliminary evidence indicates that the inexperienced student HCWs forming a strand of the UK response to Covid-19 may need additional short-term support. Short-term voluntary interventions focused on practical assistance, resilience and adaptive coping could help to minimize risk of experiencing long-term MH problems. Organizations play a key role as health worries are increased when HCWs have lower trust in protective measures and feel that information, training or support has been inadequate. Hospital managers and those in strategic roles should also anticipate long term resource problems as high-risk HCWs can experience depression and secondary health problems at least 12 months post pandemic. The role of pre-existing MH conditions is largely unknown, compromising internal validity, and suggesting an area of much needed research. Similarly, health care experience should form a more explicit focus of study designs. This is particularly pertinent in the UK due to the diverse HCW population responding to Covid-19. Researchers might consider differences in coping mechanisms between HCWs returning to the NHS, already working in the NHS, and newly qualifying HCWs.

## Data Availability Statement

The original contributions presented in the study are included in the article/Supplementary Material, further inquiries can be directed to the corresponding author/s.

## Author Contributions

SW and SG developed inclusion/exclusion criteria. SW conducted the literature search, selection of papers for inclusion, data extraction, narrative synthesis, wrote the introduction, methods, and results sections. SG conducted inter-rate reliability on papers for inclusion, quality assessment of papers, contributed to methods section write-up, and wrote the discussion. Both authors read and approved the final manuscript, and have agreed to be personally accountable for their own contributions and ensuring questions relating to accuracy or integrity of the work are appropriately investigated, resolved, and the resolution documented.

## Conflict of Interest

The authors declare that the research was conducted in the absence of any commercial or financial relationships that could be construed as a potential conflict of interest.

## Abbreviations

CHQ: Chinese Health Questionnaire
COPE: Coping Orientations to Problems Scale
DAS: Depression and Anxiety Scale
DSM: Diagnostic Statistical Manual
GAD: Generalized Anxiety Disorder Scale
GHQ: General Health Questionnaire
GMR: Global Mortality Rate
HCW: health care worker
IES-R: Impact of Events Scale-Revised
MERS: Middle East respiratory syndrome
MH: mental health
NHS: National Health Service
PHQ: Patient Health Questionnaire
PICO: population, interests, comparisons outcomes
PPE: personal protective equipment
PTSD: post-traumatic stress syndrome
REA: rapid evidence assessment
SARS: severe acute respiratory syndrome
WHO: World Health Organization.
