# Supplementary material for: Rapid Evidence Assessment of Mental Health Outcomes of Pandemics for Health Care Workers: Implications for the Covid-19 Pandemic
Source: Front Public Health. 2021 May 21;9:629236. doi: 10.3389/fpubh.2021.629236 (PMC8175907; doi:10.3389/fpubh.2021.629236)
Supplement: Supplementary file 1 [file Table_1.DOCX]

Table 1. Study characteristics and key findings

| Chan et al. (2005) | | |
| --- | --- | --- |
| Design | Cross-sectional survey | |
| Participants | 1,470 nurses from across 8 hospitals (91.7% female, 8.3% male; 13.4% high risk working in SARS wards, 9.2% moderate risk working in wards with some cases of suspected SARS, 77.4% low risk with no contact with cases of suspected SARS) | |
| Country | Hong Kong | |
| Time period | Peak period of the SARS outbreak | |
| Incident type  Aim | SARS outbreak  To examine hospital nurses’ physical and psychological health status, knowledge and risk perception of SARS, preventative measures and predictors for adoption of preventative measures. | |
| Measures | Scales designed by the authors to produce a SARS Nurses’ Survey Questionnaire (measuring self-rated health, anxiety and stress, health service utilisation, contact with SARS patients, perceptions of SARS, preventative measures, degree of hope, and demographic information) | |
| Key findings | - The three major SARS related physical symptoms reported by nurses were headaches (43.8%-56.3%), back pain (33.3%-35%), and sore throat (23.6%-31%). Significantly more nurses in the high and moderate risk groups reported suffering from headaches, myalgia, and dizziness compared to the low risk group. Significantly more nurses in the high-risk group went to an emergency department. - 68.3%-80.1% of nurses across groups reported stress associated with the SARS outbreak. Stress was significantly higher in the moderate risk group. 43.8%-58.5% also perceived home stresses, and 35%-46% perceived community stresses. 50.7% of nurses in the moderate risk group perceived that they sometimes could not cope with this stress, compared with 45.6% in the high risk and 38.7% in the low risk groups. - 87% of high risk and 80.7% of medium risk nurses believed they were likely to contract SARS, which was significantly higher than the low risk group (65%). 69.4%-75.6% believed they were likely to survive if infected. - Nurses working in the high-risk wards, those who had children, those reporting any SARS-related symptoms, and those who perceived stress from SARS were significantly more likely to adopt all specified preventative measures outside of work. | |
| Chan and Huak (2004) | | |
| Design | Cross-sectional survey | |
| Participants | 661 HCWs (548 nurses, 113 doctors; 106 had direct contact with SARS patients, 555 did not have direct contact) | |
| Country | Singapore | |
| Time period | Two months post-outbreak | |
| Incident type  Aim | SARS outbreak  To describe the psychological impact of SARS on HCWs in a regional general hospital. | |
| Measures | General Health Questionnaire; Impact of Events Scale; scale designed by the authors to measure changes in life priorities due to SARS and coping. | |
| Key findings | - Doctors were 1.6 times more likely to experience psychiatric symptoms than nurses. Single HCWs were 1.4 times more likely to experience psychiatric symptoms compares with married HCWs. - Logistic regression showed that HCWs that believed work had become more important and those that agreed support from supervisors/colleagues, and clear communication of precautionary measures had helped them to cope better were less likely to report psychiatric symptoms. - Those who perceived they were receiving support from supervisors/colleagues were least likely to report symptoms of PTSD. - There were no differences between those who had direct contact with SARS patients and those who did not and reported coping and psychiatric symptoms. | |
| Chen et al. (2006) | | |
| Design | Intervention | |
| Participants | 116 nursing staff (98.3% female, 1.7% male) | |
| Country | Taiwan | |
| Time period | Questionnaires were administered before nurses cared for SARS patients, 2 weeks after caring for SARS patients, 1 month after the prevention programme began, and 1 month after the hospital returned to normal functions. | |
| Incident type  Aim | SARS outbreak  To determine the levels of anxiety, depression, and sleep quality experienced by nursing staff before and after a SARS prevention program | |
| Measures | Zung’s self-rating anxiety scale; Zung’s self-rating depression scale; Pittsburgh sleep quality index | |
| Key findings | - Higher anxiety and depression levels were associated with lower family support, increased stress before the SARS outbreak, and not volunteering to care for SARS patients. - Anxiety and depression levels increased and sleep quality decreased when nurses began to care for SARS patients. - Both anxiety and depression levels significantly reduced after completing the prevention program and continued to decrease over time, even becoming lower than they were prior to treating SARS patients. The prevention program included a series of in-house training, detailed manpower allocation, adequate PPE, and availability of a mental health team. | |
| Chen, Wu, Yang, and Yen (2005) | | |
| Design | Cross-sectional survey | |
| Participants | 131 nurses (128 female, 3 male; 65 working in high risk units, 21 involuntarily conscripted to work in high risk units, 45 working in low risk units) | |
| Country | Taiwan | |
| Time period | Mid-May 2003 during the peak of the SARS outbreak | |
| Incident type  Aim | SARS outbreak  To explore whether nurses working during the SARS pandemic showed symptoms of distress and stress from being conscripted to work in higher risk units | |
| Measures | Impact of Events Scale; 90-item Symptom Checklist-Revised (self-report rating scale designed to measure general psychopathology associated with the constructs of somatization, obsessive- compulsive symptoms, interpersonal sensitivity, depression, anxiety, hostility, phobic anxiety, paranoid ideation, and psychoticism). | |
| Key findings | - 11% of high-risk nurses, 10% of conscripted nurses and 2% of low-risk nurses reported stress reaction syndrome. Those with stress reaction syndrome reported more severe symptoms of depression, anxiety, hostility, and somatization. - Nurses in the conscripted group reported significantly more severe symptoms of intrusion and avoidance, depression and hostility than nurses in the low-risk group. Nurses in the conscripted group also reported significantly more severe symptoms of intrusion, depression and psychoticism compared with nurses in the high-risk group. Nurses who always perceived stress reported poorer health. | |
| Chong et al. (2004) | | |
| Design | Cross-sectional survey | |
| Participants | 1257 staff from the Chang Gung Memorial Hospital, Taiwan (81.1% women, 18.9% men; 54% nurses, 11% doctors, 11% health administrators, 24% other professionals such as pharmacists, technicians and respiratory therapists); 53 responses were excluded due to incomplete answers | |
| Country | Taiwan | |
| Time period | May – June 2003 (Initial phase: 12 May – 6 June, repair phase: 7 – 27 June) | |
| Incident type  Aim | SARS outbreak  To assess SARS-related stress and its immediate psychological impact and responses among HCWs (frequency of intrusive and avoidant phenomena in response to a stressful life event and psychiatric morbidity) | |
| Measures | Impact of Events Scale (IES); Chinese Health Questionnaire (CHQ); questions designed by the researchers to gather information about exposure to SARS and working experience (risk, adverse experience, and coping); demographics | |
| Key findings | - HCWs were significantly more likely to report that their job put them at risk, work stress, fear of falling ill, being stigmatized and rejected by others, and worry about passing SARS on to family, friends, colleagues or others in the initial response compared to the recovery phase. - Most HCWs reported not being willing to take the risk of caring for patients with SARS in either phase, and believed they would have little chance of survival if infected. A greater proportion of HCWs thought of resigning in the repair rather than in initial response phase. - IES scores were significantly higher in men, technicians, HCWs with less than 2 years experience, and those exposed to SARS and not living with their family during the repair phase. - Prevalence of psychiatric morbidity was 75.3% and was associated with higher IES scores. HCWs responsible for SARS patients, especially women, manifested higher rates of psychiatric morbidity. Exposure to SARS and the repair phase were significant independent predictors of psychiatric morbidity. Role, work experience, and being quarantined had no significant effect in multiple regression analysis.   77.4% reported anxiety, 74.2% depression and poor family relationships, 69% somatic symptoms, and 52.3% sleep problems. Anxiety was more frequent in the response phase; depression and poor family relationships, somatic symptoms, and avoidance were more frequent in the repair phase. | |
| Chua et al. (2004) | | |
| Design | Questionnaire | |
| Participants | 271 healthcare workers (60% nurses, 17% ward assistants, 12% doctors) from SARS units at 2 major hospitals (60% respiratory medicine, 15% internal medicine, 15% deployed specialties) and 342 healthy control subjects (balanced for age, sex, education, parenthood, living circumstances). | |
| Country | Hong Kong | |
| Time period | During the 2003 SARS outbreak | |
| Incident type  Aim | SARS outbreak  To quantify the psychological impact of SARS on high-risk health care workers. | |
| Measures | Perceived Stress Scale; structured list of psychological effects of SARS | |
| Key findings | - HCWs and controls show similar stress levels but this was 50% more than normative value and higher than for unemployment and separation. - Perceived stress and negative psychological effects from SARS were highly significantly correlated, and 80% affirmed these effects were not attributable to other contemporaneous events. - HCWs showed significantly more positive psychological effects than control subjects (reported by 94% of HCWs), including awareness of hygiene (85%), focus on current affairs (77%), unity (51%), and awareness of danger (41%). - 89% of HCWs experienced negative effects such as tiredness (71%), worry about health (59%), and fearing social contact (46%). - HCWs who were confident about infection control (74%) had lower stress levels and fewer negative effects. Confidence about infection control was independent of education, potentially due to 100% attendance at infection control training. | |
| Corley, Hammond, and Fraser (2010) | | |
| Design | Phenomenological approach | |
| Participants | 34 nursing and medical staff from a hospital in Brisbane, Australia, completed the questionnaire and 16 participated in four small focus groups (non-probability purposeful sampling for open-ended questionnaire and opportunistic sample for focus groups) | |
| Country | Australia | |
| Time period | September 2009 | |
| Incident type  Aim | 2009 n-H1N1 pandemic  To document and describe the experiences of HCWs in ICU | |
| Key findings | Use of PPE: Perceived lack of concrete guidelines and as PPE supplies ran low, guidelines of what was considered appropriate and sufficient PPE changed. Wearing the kit was uncomfortable and made it extremely difficult to stay properly hydrated and communicate.  Infection Control: Patient isolation procedures created extra workload for staff, such as educating relatives about which PPE to use and how. Lack of protocol and recording caused substantial confusion about whether a patient was infectious or not. Staff felt they should have been permitted to wear theatre scrubs (which can be laundered in the hospital) on wards so as to not bring potentially infection-carrying uniform home with them.  Fear of contracting or transmitting the infection: Fear of contracting H1N1 was especially high in younger staff, with some refusing to care for patients. Fear was also ascribed to not wanting to infect family members, inconsistencies in PPE protocols, and running out of appropriate PPE.  Staffing Levels: Nurse to patient protocols established pre-pandemic could not always be maintained, particularly as there was a lack of support staff. Junior staff often had to adopt leadership positions but felt they received inadequate support, which threatened patient safety and felt very unfair. A substantial number of staff felt fatigued due to having to work overtime and a lack of adequate time for meal breaks.  Morale: Moral was reasonably high due to medical/nursing staff “pulling together” and supporting each other, as well as management teams taking extra measures to ensure staff felt appreciated and their hard work recognised.  Patient Care: Caring for the patients, many of whom were young, was especially emotionally difficult for staff. Patients’ families also required more emotional support from HCWs than normal. | |
| Dewar, Barr, and Robinson (2014) | | |
| Design | Phenomenological approach | |
| Participants | 22 Emergency Management Coordinators across the Victorian hospital system | |
| Country | Victoria, Australia | |
| Time period | July-October 2011 | |
| Incident type  Aim | Drawing on past experience of managing the 2009 H1N1 pandemic  To investigate pandemic influenza preparedness in acute care hospitals, and whether changes had been made to policy, planning and management efforts to counter future pandemics. | |
| Measures | Open-ended questionnaires (completed by 7 rural hospitals and 15 metropolitan hospitals) and 11 follow up interviews | |
| Key findings | - Anxiety was high among staff during the 2009 pandemic but interviewees did not observe refusal to work. Hospitals employed a variety of strategies to reduce infection exposure and staff anxiety, including creating frontline ‘flu teams’ that dealt exclusively with influenza patients to reduce contact for other staff, identifying and removing susceptible HCWs such as pregnant staff from exposure, and establishing mandatory daily update meetings for department heads that were also available to other staff. Better education both before and during the pandemic was needed to keep staff aware of protocol updates and reduce uncertainty. - Rural respondents more commonly reported maintaining stockpiles of medical equipment, PPE, medication and basic supplies than metropolitan respondents. However, rural respondents rarely reported access to extra ventilators when needed or capacity to expand patient isolation in ICUs. - Supply of essential equipment and consumables during a pandemic was raised as a concern for the health system as a whole, particularly given the sector’s move towards just-in-time supply chains. Some also identified the issue of lack of timely patient results from influenza testing. | |
| Fiksenbaum, Marjanovic, Greenglass, and Coffey (2006) | | |
| Design | Cross-sectional survey | |
| Participants | 333 nurses (315 female, 18 male) | |
| Country | Torronto, Canada | |
| Time period | March-May 2004, over six months after the hospital had returned to normal service | |
| Incident type  Aim | SARS outbreak  To test a psychosocial model of factors predicting emotional exhaustion and state anger in nurses who worked during the SARS outbreak | |
| Measures | Adapted version of Eisenberger et al.’s Survey of Perceived Organizational Support; emotional exhaustion subscale of the Maslach Burnout Inventory; State-Trait Anger Expression Inventory; scales designed by authors to measure contact with patients with SARS and time spent in quarantine, perceived SARS threat, performance feedback | |
| Key findings | - Contact with SARS patients was significantly associated with higher levels of emotional exhaustion, state anger, higher levels of perceived SARS threat, spending more time in quarantine, and perceiving more positive feedback. - Lower levels of perceived organisational support were significantly associated with increased levels of perceived SARS threat, emotional exhaustion, and state anger. - Higher levels of perceived SARS threat were significantly related to higher levels of emotional exhaustion and state anger. - Emotional exhaustion was significantly positively related to state anger. - Contact with SARS patients and time spent in quarantine were significant predictors of perceived SARS threat. - The more positive feedback nurses felt they received, the more supported they felt by their organisation. | |
| Goulia, Mantas, Dimitroula, Mantis, and Hyphantis (2010) | | |
| Design | Questionnaire | |
| Participants | 469 HCWs from University General Hospital Ioannina, Greece (nurses=209, medical staff=120, allied health staff=59, auxiliary staff=81) | |
| Country | Greece | |
| Time period | September 2009 | |
| Incident type  Aim | 2009 n-H1N1 pandemic  To assess the psychological effects (psychological distress and health worries) on HCWs responding to a pandemic | |
| Measures | General Health Questionnaire and non-validated questions regarding concerns and worried about the new H1N1 pandemic | |
| Key findings | Psychological Distress: 21% HCWs reported mild to moderate distress, and 7% reported severe psychological distress. Moderate to severe psychological distress was 2.2 times more likely for nurses and 4.5 times more likely for auxiliary HCWs, compared to medical and allied HCWs.  Perceived Infection Risk: 57% of HCWs reported being worried by the H1N1 pandemic, with auxiliary HCWs (65%), nurses (61%) and allied HCWs (56%) the most worried and medical staff the least worried (36%) (sig.). HCWs reported moderately high concern about being infected by H1N1. Concerns were concentrated around risk of infecting family and friends, as well as the effect of the disease on their own ability to perform their duties.  Preparedness: Over 50% of HCWs thought their ward/department was well prepared for the pandemic, however nurses were significantly less confident about this than all other HCW groups.  Worry and Behaviour: 7% of HCWs restricted social interactions because they perceived their work environment to be dangerous. 4% believed family/friends were avoiding them because of their work. The strongest significant predictors of worry were: believing infection would have major health consequences, believing infection would be difficult to treat, and being an auxiliary HCW. Perceived sufficiency of information regarding H1N1 prognosis was the most substantial negative predictor. | |
| Grace, Hershenfield, Robertson, and Stewart (2005) | | |
| Design | Cross-sectional survey | |
| Participants | 193 physicians across three large teaching hospitals that provided care to SARS patients | |
| Country | Toronto, Canada | |
| Time period | During the 2003 SARS outbreak | |
| Incident type  Aim | SARS outbreak  To empirically assess psychosocial and occupational impacts of SARS on physicians working in hospitals where infected patients are being treated. | |
| Measures | Questionnaire (non-validated, measuring health status, attitudes and perceptions toward SARS; SARS related coping methods, concerns and symptoms; effects on personal relationships and changes to work resulting from the SARS outbreak) | |
| Key findings | - 5.7% felt pressured to work with SARS patients, 8.8% considered not going into work to avoid SARS, and 9.3% reported that the outbreaks had caused them to evaluate their career choice. - The majority of physicians perceived that their work had been seriously affected by SARS outbreaks, including interruptions to teaching and education (84.5%), unwillingness of patients to attend outpatient clinics (79.8%), infection control precautions (77.2%), inability to see outpatients (71%), inability to perform regular activities (51.8%), interruptions to research (51.8%), new involvement in SARS related work (8.8%), and inability to enter work due to symptoms (4.1%). - 18.1% reported experiencing new SARS-like symptoms, and 10.9% reported attending work regardless. 16.1% reported experiencing non-SARS symptoms, however 48.4% of these physicians in fact had symptoms listed on the SARS screening protocol. - 18.1% reported experiencing new distressing psychological symptoms that they attributed to working during the SARS outbreaks. There were no significant sex differences in the reporting of new psychological symptoms but rate of psychological distress was significantly higher among physicians providing direct care to SARS patients than those not providing direct care. - 35.8% felt they had been stigmatized because others knew they had potentially been exposed to a SARS patient, and this was higher in physicians providing direct care to SARS patients. Caucasian/white physicians perceived significantly less effect of their ethnocultural background on people’s perceptions of them than did Asian physicians. 9.3% reported that SARS had affected their relationships with their family and friends, either by stigmatization or avoidance or decreases in contact with others. - 27.5% were concerned about spreading SARS to a family member, specifically young children, elderly parents or partners, or pregnant family members. 6.2% had changed living or sleeping arrangements because of SARS. Physicians who provided direct care to a suspected or probable SARS patient were significantly more likely to be concerned about spreading SARS to family and more likely to change living arrangements. - Three ways of coping were reported: psychosocial techniques such as enlisting social support and engaging in positive health behaviours, active techniques based on the best available scientific evidence, or avoidant strategies to impede contracting or spreading the infection. - Physicians’ main concerns were decreased ability to care for non-SARS patients during outbreaks and personal loss of income. However, 40% also noted increased collegiality and teamwork. | |
| Hawryluck et al. (2004) | | |
| Design | Cross-sectional survey | |
| Participants | 129 people who had been quarantined (68% HCWs, 31% members of the public) | |
| Country | Toronto, Canada | |
| Time period | Completed by participants at the end of their quarantine (10-66 days) | |
| Incident type Aim | SARS outbreak  To examine the psychological effects of quarantine on people in Toronto. | |
| Measures | Impact of Event Scale-Revised; Centre for Epidemiologic Studies-Depression Scale; scales designed by the authors to measure knowledge and understanding of the reasons for quarantine, adherence to infection control directives, and source of this knowledge. | |
| Key findings | - Those notified to quarantine by the media or their workplace had a better understanding of the reasons than those notified by healthcare providers or public health units. - Perceived lack of information on infection control measures was associated with frustration with health institutions and public health officials at being difficult to contact, disappointment at lack of support, and anxiety abut lack of information about modes of transmission and prognosis. - HCWs more frequently reported receiving adequate information than members of the public but there were no differences between NCWs and non HCWs in terms of adherence to recommended infection control measures. - PTSD symptoms and depressive symptoms were correlated. - Age, education level, HCW status, being married, and having children were not associated with PTSD or depressive symptoms. However, spending longer in quarantine was associated with both PTSD and depressive symptoms. - 59% were worried about infecting their family, and 51% felt stigmatized. All respondents reported feeling socially isolated. | |
| Ho, Kwong-Lo, Mak, and Wong (2005) | | |
| Design | Cross-sectional survey | |
| Participants | 179 HCWs (126 female, 53 male), sample 1 - 82 HCWs working in hospitals with patients with SARS, sample 2 – 97 HCWs that were recovering from SARS | |
| Country | Hong Kong | |
| Time period | 5^th^ April-5^th^ May 2003 during peak of infection (sample 1) and August 2003 (sample 1) | |
| Incident type Aim | SARS outbreak  To examine fear of SARS among HCWs who had and had not contracted the disease. | |
| Measures | Sample 1 received scales designed by the authors to measure fear related to SARS and SARS self-efficacy; sample 2 received Chinese Self-Efficacy Scale and Chinese Impact of Events Scale-Revised. | |
| Key findings | - HCWs at the peak of infection reported more fear related to infection than HCWs recovering from infection. HCWs recovering from infection were more concerned about death, discrimination, quarantine, and health problems arising as side effects of SARS treatment. - HCWs that felt less able to comply with infection control measures had higher fear related to change in workload and duties as a result of SARS. - In HCWs recovering from infection, SARS-related fears were related to perceived self-efficacy and PTSD. | |
| Koh et al. (2005) | | |
| Design | Questionnaire | |
| Participants | 10,511 healthcare workers from 9 major healthcare institutions in Singapore | |
| Country | Singapore | |
| Time period | During the 2003 SARS pandemic | |
| Incident type Aim | SARS outbreak  To understand the impact of the SARS pandemic on HCWs’ perceptions of risk, and impact on personal and working life. | |
| Measures | Impact of Events Scale and non-validated scales measuring individual characteristics, perception of exposure to SARS, risk of infection and impact on personal and work life | |
| Key findings | - 76% were afraid of falling ill with SARS and 66% reported feeling at greater risk of exposure to SARS. Significant factors independently associated with greater perception of risk were: hospitals treating SARS patients, occupation (doctors, nurses and ambulance crew felt at greater risk than physiotherapists and healthcare attendants), daily exposure to SARS patients, and high IES scores. Gender, marital/parental status and number of years’ experience were not significant factors. - 82% were concerned about inadvertently spreading the disease to family, friends and colleagues. 69% thought people close to them were worried they might become infected through them. 87% felt that people close to them were worried for the HCWs’ health. - 49% felt people avoided them because of their job, and 31% that people avoided their family members because of their job. HCWs with higher IES scores were more likely to feel stigmatized. - 82% felt appreciated by their hospital/clinic/employer, and 77% felt appreciated by society. Those least involved in direct patient care (health attendants/cleaners/administrative staff) were more likely to feel appreciated by society than doctors and nurses. - 56% felt more stressed at work, 53% experienced increased workload, 54% had to undertake work they normally would not do, and 36% had to work overtime. Predictors of increased stress included: SARS-affected hospitals, daily exposure to SARS patients, occupation (nurses), married with children, and high IES scores. - 96% felt preventative measures implemented at work were generally effective, and 95% were satisfied with the explanation of their necessity. 93% felt policies and protocols were clear and 90% felt these were implemented quickly enough. 92% felt that staff adhered to recommended measures consistently, and 72% reported little difficulty doing so. Doctors found measures easiest to adhere to in comparison to nurses, cleaners, pharmacists, health attendants, and administrative staff. | |
| Lai et al. (2020) | | |
| Design | Cross-sectional survey | |
| Participants | 1257 healthcare workers (493 physicians 39.2%, 764 nurses 60.8%) from across 34 hospitals equipped with fever clinics or wards for COVID-19. 20 hospitals in Wuhan (10 designated by the local government to treat covid-19 and 10 non-designated), 7 hospitals in other regions of Hubei province, and 7 hospitals from 7 other provinces with a high incidence of covid-19 (1 hospital from each province) were included. | |
| Country | China | |
| Time period | 29^th^ January to 3^rd^ February 2020 – during covid-19 pandemic | |
| Incident type  Aim | Covid-19 pandemic  To assess the magnitude of mental health outcomes and associated factors among healthcare workers treating patients exposed to COVID-19 in China. | |
| Measures | Patient Health Questionnaire; Generalized Anxiety Disorder Scale; Insomnia Severity Index; Impact of Events Scale-Revised | |
| Key findings | - 50.4% had symptoms of depression, 44.6% of anxiety, 34% of insomnia, and 71.5% of distress. - Nurses, women, frontline workers, and those based in Wuhan (most effected region) reported more severe symptom levels of depression, anxiety, insomnia, and distress. Working on the frontline was an independent risk factor for depression, anxiety, insomnia, and distress. - HCWs working in secondary hospitals were more likely to report severe symptoms of depression, anxiety and insomnia than tertiary hospitals. - After controlling for confounders, being a woman and having an intermediate professional title were associated with severe symptoms of depression, anxiety, and distress. | |
| Lancee, Maunder, and Goldbloom (2008) | | |
| Design | Structured Clinical Interview for DSM-IV (SCID) | |
| Participants | 133 HCWs across 9 hospitals in Toronto, Canada | |
| Country | Canada | |
| Time period | October 2004 – September 2005 (one to two years following the resolution of the outbreak) | |
| Incident type  Aim | SARS outbreak  To assess the prevalence of new onset mental illness in HCWs since the resolution of SARS | |
| Measures | Clinical-Administered PTSD Scale; Structured Clinical Interview; Impact of Events Scale; Kessler Psychological Distress Scale; Emotional Exhaustion Scale of the Maslach Burnout Inventory; self-report of smoking, drinking alcohol, non-prescription drugs or other activities that would interfere with work or relationships | |
| Key findings | - 4% of HCWs experienced major depressive episodes and 2% experienced new-onset PTSD. Onset of any other Axis I disorder was 7%. - There was a significant, albeit small, association between previous psychiatric history and novel onset of Axis I. Protective effects against novel onset were observed for number of years’ experience and perception of being adequately trained and supported. | |
| Lee et al. (2005) | | |
| Design | Questionnaire, two focus groups, interviews | |
| Participants | 26 female nurses working in a SARS team (10 participated in focus group 1, 22 in focus group 2, 21 were interviewed in small groups, 26 completed the questionnaire) | |
| Country | Taiwan | |
| Time period | May-June 2003 as hospitals were returning to normal functioning | |
| Incident type  Aim | SARS outbreak  To identify staff stress and coping strategies among a SARS team of nursing staff during the outbreak | |
| Measures | Focus groups and interviews that focused on SARS experiences, psychological conflicts and stresses, coping strategies, and preventative and intervening measures. This was used to design questionnaires that measure (a) immediate reactions to the mission; (b) major stressors inherent in caring for SARS patients; (c) effective measures to reduce stress; (d) coping strategies; (e) motivators to join future missions; and (f) evaluation of psychiatric services. | |
| Key findings | - 65-69% held a positive attitude toward their assignment and 12-31% felt mixed feelings of anxiety, fear, depression and loss of control, stating that it was too much to expect them to risk their own lives and wellbeing to be “heroines”. - The majority reported stresses related to colleagues (92%), patients (89%) and family (89%). Stress was exacerbated by changes n infection control measures (92%), documentation process (81%), and increases in casualties (92%). - 81% were also concerned about the lack of knowledge about the virus. 81% felt they were not armoured with appropriate PPE and this was threatening their wellbeing. Wearing PPE hindered work and caused discomfort (89%), compromising quality of care (73%). - 77% reported the loss of the head nurse due to SARS as a major stressor to them. - Conflicts were reported with doctors who interacted with patients and their families in a negative manner, increasing the burden on nurses. - All nurses felt the psychiatric services and staff support were effective in helping them to manage stress but that they needed to be more flexible, informal and relaxing to improve with scheduling. Nurses commented that more individual counselling to focus on personal issues would be helpful, running sessions in groups smaller than nine, ad shortening time to less than 50 minutes, along with continuous delivery as HCWs were still experiencing secondary trauma. - 96% also felt that having enough rest and time off, and appropriate work shifts helped them to reduce stress and restore energy, and that enforcement of stringent infection control, adequate PPE supplies, and the education program demonstrated strong support from hospital administration. 92% also felt senior nurses’ mentoring junior nurses improved morale. - Coping strategies used by at least 96% of nurses included adopting more active personal protective measures, learning more about the disease, and engaging in health-promoting behaviours. Team members frequently met for debriefs after work and exchanged information regularly. More than three quarters of nurses engaged in recreational activities and found support in speaking with families and friends over the telephone, sending pictures of themselves in PPE to reassure family and friends. 62% avoided watching news about SARS on TV, which had been disturbing, unfair, and imposed unnecessary pressure on them. | |
| Lin, Wu, Chang, Chan, and Yang (2007) | | |
| Method | Questionnaires | |
| Participants | 92 medical staff (doctors and nurses) | |
| Country | Taiwan | |
| Time period | The month after the 2003 SARS outbreak ended. | |
| Incident type  Aim | SARS outbreak  To determine the influence of SARS on the psychological status, including PTSD symptoms, of staff in the emergency department (compared emergency department staff in the high-risk ward with the psychiatric ward staff in the medium-risk ward). | |
| Measures | Davidson Trauma Scale-Chinese; Chinese Health Questionnaire | |
| Key findings | - 4.3% considered SARS a very serious stress in their life, 41.3% considered it a serious stress, and 47.8% considered it a mild stress. - 19.3% reported DTS-C scores >40, which indicates PTSD is highly suspected. There was no difference in age, sex, marital status, workload, number of children, or number of family members in those with scores above and below 40. - HCWs with DTS-C scores above 40 were more likely to rate stress caused by SARS as serious than in those with DTS-C scores below 40. - 47.78% of staff had CHQ-12 scores >3, indicating minor psychiatric morbidity. There was no significant difference by sex, age, marital status, work load, number of children, number of family members, history of physical and mental illness, history of quarantine due to suspected SARS and the self-observation about the severity of the stress caused by SARS. - Average DTS-C scores were significantly higher in HCWs in high-risk compared to the medium-risk departments. - HCWs in the high-risk department experienced PTSD symptoms ‘‘acting and feeling as if the trauma were recurring’’ and ‘‘irritability’’ more severely and more often, and experienced more difficulty in ‘‘getting along with the family or friends’’ than HCWs in the low risk department. | |
|  |  |  |
| Liu et al. (2012) | | |
| Design | Cross-sectional survey | |
| Participants | 549 hospital workers (75.2% female, 24.8% male) | |
| Country | China | |
| Time period | 2006, 3 years after the SARS outbreak | |
| Incident type  Aim | SARS outbreak  To examine post-outbreak levels of depressive symptoms and the relationship with types of outbreak event exposures experienced. | |
| Measures | Centre for Epidemiological Studies Depression Scale; Impact of Events Scale-Revised; scales designed by the authors to measure exposure to SARS (work exposure, quarantining, having a close friend or relative contract SARS) and other exposure to traumatic events prior to the SARS outbreak, and perceptions of SARS-related risk | |
| Key findings | - Approximately 14% reported moderate levels of depressive symptoms and 8.8% reported high levels of depressive symptoms. - Bivariate analysis indicated that higher levels of depressive symptoms were associated with younger age, being single, work exposure, and being quarantined. Exposure to other traumatic events prior to the SARS outbreak was also associated with depressive symptoms, whereas altruistic acceptance of SARS-related risk was associated with lower depressive symptoms. Experiencing high levels of posttraumatic stress symptoms during and after the outbreak was also associated with higher levels of current depressive symptoms. Those currently working in high-stress jobs also tended to report higher current depressive symptoms. Multinomial regression analysis identified these same relationships. | |
| Liu et al. (2020) | | |
| Design | Cross-sectional survey | |
| Participants | 512 HCWs (84.57% female, 13.43% male) | |
| Country | China | |
| Time period | 10^th^-20^th^ February 2020, as hospitals in China move into recovery | |
| Incident type  Aim | Covid-19  To identify the factors influencing HCW anxiety in China during the covid-19 outbreak. | |
| Measures | Zung Self-rating Anxiety Scale; scale developed by authors to measure contact with patients with covid-19, whether HCWs have symptoms of covid-19, adherence to control measures, and psychological treatment need. | |
| Key findings | - Anxiety was significantly higher in HCWs who had directly treated confirmed cases. - Univariate analysis and multivariable linear regression shows that increased anxiety is associated with direct treatment, residence in Hubai province, and having a suspected infection. - Gender, age, education, marital status, location, satisfaction with effectiveness of community prevention measures and need for psychological counselling did not increase anxiety scores. | |
| Lu, Shu, Chang, and Lung (2006) | | |
| Design | Cross-sectional survey | |
| Participants | 127 HCWs that had contact with or cared for patients with SARS (74 female, 53 male; 49 nurses, 24 physicians, 54 medical technicians, respiratory therapists or emergency attendants) | |
| Country | Taiwan | |
| Time period | July 2003-March 2004, as the hospital returned to normal service, through to eight months later | |
| Incident type  Aim | SARS outbreak  To explore the impact of SARS on HCWs in Taiwan | |
| Measures | Chinese Health Questionnaire; Eysenck Personality Questionnaire; Parental Bonding Instrument | |
| Key findings | - 17.3% demonstrated psychiatric morbidity on the CHQ. Multiple linear regression analysis also showed no significant differences in mental health and age, gender, educational level, marital status and occupation. - Neuroticism was associated with negative mental health outcomes. Participants that lacked maternal caring or who were overprotected by their mother also reported worse mental health outcomes. | |
| Lung, Lu, Chang, and Shu (2009) | | |
| Design | Cross-sectional survey | |
| Participants | 127 HCWs that had contact with or cared for patients with SARS (74 female, 53 male; 49 nurses, 24 physicians, 54 medical technicians, respiratory therapists or emergency attendants) | |
| Country | Taiwan | |
| Time period | July 2003-March 2004 (after the hospital had returned to normal function) and a year later | |
| Incident type  Aim | SARS outbreak  To assess the psychological impact of SARS on HCWs | |
| Measures | Chinese Health Questionnaire; Eysenck Personality Questionnaire; Parental Bonding Instument; followed by completing the CHQ again a year later | |
| Key findings | - A year later, 15.4% demonstrated psychiatric morbidity on the CHQ, which is lower than the 24% estimated prevalence amongst the general population in Taiwan. 30.9% reported life or job stresses. - Multiple linear regression analysis showed no relationship between participants’ mental health and age, gender, marital status and occupation at either of the two time points. However, during the 12-month follow up, lower education and higher neuroticism were associated with poorer mental health. Daily-life stressors during the year following the SARS outbreak also negatively impacted mental health. - Mental health worsened for HCWs who were overprotected by their mother before the age of 16 when encountered with daily-life stressors. Neurotic personality also had a negative impact on mental health at both time points. - Physicians reported more somatic symptoms than nurses. | |
| Marjanovic, Greenglass, and Coffey (2007) | | |
| Design | Questionnaire | |
| Participants | 333 nurses (95% female) | |
| Country | Canada | |
| Time period | March to May 2004 | |
| Incident type  Aim | After the 2003 SARS outbreak in Canada  To examine the relationship between psychosocial variables, working conditions, coping methods and distress in response to the SARS crisis in Canada. | |
| Measures | Emotional exhaustion subscale of Maslach Burnout Inventory – General; state anger subscale of STAXI; scales created by the authors to measure avoidance, vigor and equipment; Survey of Perceived Organisational Support | |
| Key findings | - Emotional exhaustion was significantly positively correlated to state anger, avoidance behaviour, contact with SARS patients, and time spent in quarantine; and significantly negatively correlated to vigor, organisational support, and trust in equipment / infection control initiatives. - State anger was significantly positively correlated to avoidance behaviour, contact with SARS patients, and greater time in quarantine; and negatively related to vigor, organisational support, and trust in equipment / infection control initiatives. - Avoidance coping was positively correlated to time spent in quarantine and negatively related to vigor, organizational support, and trust in equipment/infection control initiatives. Avoidance was only marginally correlated to contact with patients with SARS - even when confronted with more contact with SARS patients most nurses continued to implement disease control initiatives and kept working. - Less time spent in quarantine and higher levels of organizational support predicted lower avoidance. - Emotional exhaustion was predicted by greater contact with SARS patients, and lower vigor and trust in equipment / infection control initiatives. - Of the psychosocial variables, vigor was the most influential, predicting all three criterions. Organizational support was negatively related to avoidance and state anger, while trust in equipment/infection control initiatives was negatively related to emotional exhaustion and state anger. Of the working conditions variables, greater time spent in quarantine was predictive of higher levels of avoidance, and state anger, whereas greater contact with SARS patients was only predictive of greater emotional exhaustion. | |
| Matsuishi et al. (2012) | | |
| Design | Cross-sectional survey | |
| Participants | 1625 HCWs from across three tertiary teaching hospitals in Kobe that had accepted H1N1 cases (1228 female, 397 male; 218 doctors, 864 nurses, 543 other) | |
| Country | Japan | |
| Time period | Late June 2009, just as the hospitals were returning to normal functioning | |
| Incident type  Aim | H1N1 outbreak  To investigate the psychological impact of the H1N1 pandemic on hospital workers and how it was affected by the characteristics of the hospital, gender, age, job, and work environment. | |
| Measures | Impact of Events Scale; scale designed by the authors to measure stress associated with H1N1 and sociodemographic characteristics. | |
| Key findings | - Workers in their 20s were significantly more anxious about infection than workers in their 40s, 50s and 60s. Nurses and other roles were significantly more anxious about infection than doctors. Workers in high-risk environments were significantly more anxious about infection than workers in low-risk environments. - Workers in their 50s were significantly more exhausted than workers in their 20s.Nurses were significantly more exhausted and reported higher workload than doctors. Workers in high-risk environments were significantly more exhausted and reported higher workloads than workers in low risk environments. - Workers in their 20s felt significantly less protected than workers in their 40s, 50s and 60s. Nurses and other roles felt significantly more protected than doctors. - Post-traumatic stress symptoms were significantly greater in workers in high-risk than low-risk departments, and in nurses and other roles compared with doctors. | |
| Maunder et al. (2003) | | |
| Design | Ethnographic approach | |
| Participants | Vice-President of Nursing, Program Director of Nursing, Chair of the Medical Advisory Council, Director of Community Health Programs, mental health professionals attending patients with and without SARS (2 consultation–liaison psychiatrists, a psychiatric clinician nurse specialist and a social worker) and a psychiatrist who met with HCWs individually to provide support at their request. | |
| Country | Toronto, Canada | |
| Time period | 3^rd^-13^th^ April 2003, at the peak of the SARS outbreak. | |
| Incident type  Aim | SARS outbreak  To describe the psychological and occupational impact of the SARS outbreak within a large hospital during the first four weeks and the subsequent administrative and mental health response. | |
| Measures | Unstructured interviews, clinical observations of patients with and without SARS also receiving care from the same health workers and services. | |
| Key findings | - Staff were adversely affected by fear of contagion and of infecting family, friends and colleagues. - Caring for HCWs as patients and colleagues was emotionally difficult. Uncertainty (which was increased by modification to infection control procedures and public health recommendations day-by-day, and sometimes hour by hour) and stigmatization (some staff avoided telling people they were hospital workers) were prominent themes for both staff and patients. - Staff felt isolated because their usual forms of work support were discouraged, including no staff meetings, or face-to-face meetings with colleagues. Financial burdens increased as staff were unable to work across multiple health facilities, and it also left them worried that they were vectors of the disease. There were also worries about staffing issues due to HCWs being quarantined. - Although there were incidents of professional and nonprofessional staff refusing to care for patients with SARS in respiratory isolation on general medical floors, there was no refusal by nurses on the SARS unit. Staff attributed this to feeling confident about being well equipped, maximally protected by isolation precautions and well supported in the hospital. | |
| Maunder et al. (2004) | | |
| Design | Cross-sectional survey | |
| Participants | 1,557 HCWs across three hospitals (74.6% female, 25.4% male; 26.6% nurses, 7.5% clerical, 7.5% research laboratory, 7.4% physicians, 7.2% administration, 6.8% clinical laboratory, 3.1% social work, 2.9% occupational and physiotherapy, 2.8% pharmacy, 1.7% clinical assistant, 1.7% housekeeping, 2.1% other clinical, 5.1% nonclinical, 16.6% other), 1,300 from hospitals that treated SARS patients, 257 from a hospital that did not treat SARS patients) | |
| Country | Toronto, Canada | |
| Time period | May and June 2003 as hospitals were returning to normal functioning | |
| Incident type  Aim | SARS outbreak  To measure psychological stress in hospital workers and factors that may have mediated acute traumatic responses. | |
| Measures | Impact of Events Scale; scales designed by the authors to measure attitudes to the outbreak | |
| Key findings | - Higher IES score was associated with SARS patient contact and being a nurse. - Results of regression analysis show that the associations between both contact with patients with SARS and being a nurse and increased psychological distress are mediated by health fear, social isolation, and job stress. | |
| Maunder et al. (2006) | | |
| Design | Questionnaire | |
| Participants | 769 healthcare workers from across 9 Toronto and 4 Hamilton hospitals (Hamilton had same protocols and screenings but no SARS patients) that treated SARS patients (73.5% nurses, 8.3% clerical staff, 2.9% physicians, and 2.3% respiratory therapists) | |
| Country | Toronto, Canada | |
| Time period | 13-26 months after the outbreak in Toronto | |
| Incident type  Aim | SARS outbreak  To examine the long-term psychological and occupational effects of SARS on healthcare workers. | |
| Measures | Impact of Events Scale; Kessler Psychological Distress Scale; emotional exhaustion scale of Maslach Burnout Inventory; scales designed to measure SARS related perception of stigma and interpersonal avoidance, adequacy of training, protection, support, job stress | |
| Key findings | - Toronto HCWs reported significantly higher levels of burnout, psychological distress, and posttraumatic stress than Hamilton. Toronto HCWs reported reducing patient contact and hours of healthcare work since the SARS outbreak, along with increased substance use and other traits interfering with functioning, and more days off work. They were twice as likely as Hamilton HCWs to be experiencing multiple problems. - Burnout and posttraumatic stress were significantly predicted by maladaptive coping (e.g. avoidance, hostile confrontation, self-blame, attachment anxiety), perceived adequacy of training, and protection and support. - Psychological distress was predicted by maladaptive coping and attachment anxiety but healthcare experience had a protective effect. - Duration of post-SARS perceived risk was significantly positively associated with maladaptive coping and perceived adequacy of training, protection, and support. Prevalence of multiple adverse outcomes increased with longer duration of perceived risk. | |
| McAlonana et al. (2007) | | |
| Method | Questionnaires | |
| Participants | 2003 - 176 healthcare workers (doctors, nurses, healthcare assistants) who practiced respiratory medicine (106 high risk) and psychiatric in-patient healthcare workers (70 low risk) from two acute care Hong Kong general hospitals. 2004 – 184 (71 high risk, 113 low risk) | |
| Country | Hong Kong | |
| Time period | At the peak of the outbreak in 2003 and a year later | |
| Incident type  Aim | SARS outbreak  To assess the immediate and sustained psychological health of healthcare workers who were at high risk of exposure during the SARS outbreak. | |
| Measures | Time point 1: Perceived Stress Scale; Time point two: Perceived Stress Scale, Depression and Anxiety Scale, Impact of Events Scale-Revised | |
| Key findings | - In 2003, high-risk HCWs had elevated stress levels that were not significantly different from low-risk HCW control subjects. By 2004, stress levels in the high-risk group had increased and were significantly higher than low-risk HCW control subjects, whose stress levels had decreased. - High-risk HCWs reported a significantly higher percentage of negative responses, including fatigue, poor sleep, worry about health, and fear of social contact, despite their confidence in infection-control measures. - In 2004, depression and anxiety were significantly higher in high risk than in low risk workers. Stress levels in the high-risk group were associated with higher depression, anxiety, and posttraumatic stress scores. Posttraumatic stress scores were a partial mediator of the relation between the high risk of exposure to SARS and higher perceived stress. - Among high-risk HCWs, perceived stress was greater in men than women. Among both high and low risk HCWs, there was no difference between doctors, nurses and other HCWs. Mediation analysis indicated that posttraumatic stress owing to the impact of the SARS outbreak could account for the difference in perceived stress between high-risk and low-risk health care workers. | |
| Nickell et al. (2004) | | |
| Method | Questionnaires | |
| Participants | 2001(78.8% female) healthcare professionals (33.1%), nurses (25.6%), doctors (9.3%) and non patient care roles such as administration, food services, research maintenance (31.9) working at Sunnybrook and Women’s College Health Sciences Centre (principal tertiary referral hospital). | |
| Country | Toronto, Canada | |
| Time period | 10^th^-22^nd^ April 2003, during the outbreak | |
| Incident type  Aim | SARS outbreak  To investigate the psychosocial effects associated with working in a hospital environment during the peak of the SARS outbreak. | |
| Measures  Key findings | General Health Questionnaire; scales designed by authors to measure SARS concern, precautionary measures, personal well-being, and demographics   - Two-thirds reported SARS-related concern for their own or their family’s health. Nurses were most and doctors were least frequently concerned. - 29% scored above the threshold on the GHQ-12, indicating probable emotional distress; the rate among nurses was 45%, compared with 33.3% in other healthcare professionals, 17.4% in doctors, and 18.9% in staff not working in patient care. - Masks were reported to be the most bothersome infection control precaution, due to physical discomfort (92.9%). - Logistic regression analysis identified that increased concern for personal or family health was predicted by greater perceived risk of death from SARS, living with children, personal or family lifestyle affected by outbreak, and being stigmatized because of working in a hospital. - Four factors were identified as significantly associated with emotional distress: being a nurse, part-time employment status, lifestyle affected by SARS outbreak and ability to do one’s job affected by the precautionary measures. - 58% also reported at least one positive effect, including increased awareness of disease control (41.1%), a learning experience (26.4%), and an increased sense of togetherness and cooperation (23.8%). | |
| Phua, Tang, and Tham (2008) | | |
| Design | Cross-sectional survey | |
| Participants | 141 HCWs based in an acute general hospital that was closed to all admissions except SARS cases during the outbreak (111 female, 30 male; 83 nurses, 58 physicians) | |
| Country | Singapore | |
| Time period | November 2003, six months after the end of the outbreak | |
| Incident type  Aim | SARS outbreak  To examine the coping strategies adopted by emergency department HCWs who cared for patients infected with SARS. | |
| Measures | COPE scale (Coping Orientation to Problems Experienced); Impact of Events Scale; General Health Questionnaire | |
| Key findings | - Respondents reported significantly greater use of adaptive (emotion and problem focused) coping strategies than less useful strategies (denial, disengagement, use of drugs and alcohol). - Physicians were significantly more likely to use humour as a coping strategy and planning compared with nurses. There were no significant differences in emotion focused, problem focused or less useful strategies between roles. - Filipino HCWs were significantly more likely to use emotion-focused coping and to turn to religion to help them cope than non-Filipino HCWs. - 17.7% reported IES scores indicative of psychiatric morbidity. Those with higher IES scores were significantly more likely to report all three coping responses. - 18.8% reported GHQ scores indicative of psychiatric morbidity. Those with higher GHQ scores reported higher use of less-useful coping responses. Nurses were significantly more likely to report higher IES scores | |
| Poon et al. (2004) | | |
| Design | Questionnaire | |
| Participants | 1926 HCWs working in a regional hospital in Hong Kong (42.2% nurses, 18.1% supporting staff including healthcare assistants, technicians, workmen and transport workers, 11.9% administrative staff, 10.7% allied health workers, 7.3% doctors, and 9.7% from an unknown staff group. | |
| Country | Hong Kong | |
| Time period | Late May to early June 2003, just after the peak of the SARS outbreak but before Hong Kong was declared SARS free. | |
| Incident type  Aim | SARS outbreak  To identify anxiety levels among front-line health care workers during the 2003 ARS outbreak | |
| Measures | STAXI; emotional exhaustion dimension of the Maslach Burnout Inventory; scales designed by authors to measure level of contact with patients with SARS, use of PPE, psychological and somatic symptoms, disruption of usual routines, satisfaction with interim arrangements and support from friends, family and colleagues, perception and knowledge of SARS | |
| Key findings | - Anxiety was significantly higher among staff that had had contact with patients with SARS than among those who had not. Mean anxiety levels were significantly higher among workmen, health care assistants, and nurses than among administrative staff controls or doctors. - Anxiety was significantly positively correlated with burnout and with discomfort from wearing protective gear. - Compared with controls, front-line HCWs experienced greater discomfort from wearing protective gear, used more of it, and wore more protective gear outside of work, had more burnout symptoms, experienced more prejudice from others, were more worried about contracting SARS and cross-infecting family members, and felt at greater risk of contracting, becoming permanently disabled or dying from the disease. - Front-line HCWs also felt more encouraged and greater solidarity with fellow HCWs than did controls. A larger proportion took showers before going home, stayed away from home, and were discontent about the government’s and the health authority’s handling of the crisis. - Differences in working environment, such as use of protective equipment, nature of work (i.e. extent of patient contact), and availability of supportive measures (e.g. access to and communication of information), may account for factors contributing to differences in anxiety levels. | |
| Robertson, Hershenfield, Grace, and Stewart (2004) | | |
| Method | | Interviews |
| Participants | | 10 HCWs who were quarantined due to exposure to SARS |
| Country | | Toronto, Canada |
| Time period | | July 2003 when infection-control restrictions were being downgraded in Toronto healthcare facilities. |
| Incident type  Aim | | SARS outbreak  To examine the psychosocial effects of being quarantined because of exposure to SARS on healthcare workers. |
| Measures | | Semi-structured qualitative interviews (8 telephone, 2 face-to-face) |
| Key findings | | Loss: Knowing someone who contracted SARS heightened workers’ anxiety and fear. Quarantine was likened to prison. HCWs experienced loss of intimacy and social contact that resulted in physical and psychological isolation. Stress was placed on the entire family due to changes in roles and routines (e.g. partner needing to look after children, drop off at school, do shopping) because the other had to remain quarantined at home. Children were also frightened, and it was difficult to explain the situation without inducing more fear. Spouses were also socially isolated as they were unable to be in physical contact. HCWs experienced stigma from neighbours and friends and felt angry about it, even after the quarantine had ended. HCWs were worried their children would be stigmatized by association.  Duty: HCWs accepted that there was an inherent risk and quarantine was a necessity to protect others from further infection. None of the HCWs refused to perform their duties but there was trepidation, fear and anxiety associated with the risk of contracting SARS.  Conflict: There was conflict between duty and worry about infecting family and friends that they considered to be vulnerable. This conflict caused feelings of guilt, fear, anxiety and remorse. There was also conflict between the high-risk HCWs that continued working and ‘non-essential’ staff who remained at home and were paid. However, there was camaraderie with staff working and facing the situation together providing social support. HCWs were angry about the spread of SARS and lack of, or conflicting information given by management and public health authorities. Some felt the spread could have been curtailed if management had listened to their concerns, rather than being slow to implement vigilant safety precautions. Lack of information about how to minimize infection at home contributed to fear of contaminating family members and increased their sense of personal danger. This added to frustration directed toward management and public health authorities. |
| Sin and Huak (2004) | | |
| Design | | Cross-sectional survey |
| Participants | | 47 HCWs (18 physiotherapists, 13 occupational therapists, 3 speech therapists, 13 support staff) |
| Country | | Singapore |
| Time period | | Two months after the outbreak |
| Incident type  Aim | | SARS outbreak  To examine the psychological impact of the SARS outbreak on HCWs in a rehabilitative services department of a general hospital two months after the outbreak. |
| Measures | | General Health Questionnaire; Impact of Events Scale; scale designed by the authors to measure changes in life priorities and factors that had helped HCWs to cope |
| Key findings | | - 23.4% experienced severe psychiatric symptoms and 12.8% had symptoms of PTSD. There were no significant differences between discipline, marital status and age group. - Factors that helped HCWs to cope were predominantly having support from colleagues, taking precautionary measures, and getting clear directives and SARS disease information. - Staff that reported work becoming more important to them were less likely to report severe psychiatric symptoms. |
| Styra et al. (2008) | | |
| Design | | Questionnaire |
| Participants | | 248 healthcare workers (173 nurses; 86% female) – 88 were from low-risk units and 160 were from high-risk units (56% of these had daily contact with SARS patients). |
| Country | | Toronto, Canada |
| Time period | | 16^th^ June-9^th^ July 2003, after the peak of the SARS outbreak but before Canada was declared SARS free. |
| Incident type  Aim | | SARS outbreak  To quantify the psychological effects of working in a high-risk unit during a SARS outbreak. |
| Measures | | Impact of Event Scale-Revised; scales designed by authors to measure attitudes toward the SARS crisis (perception of personal risk, perception of their risk to others, confidence in infection control measures, confidence in information received regarding SARS, impact on personal life, impact on work life, and depressive affect) |
| Key findings | | - HCWs in the high-risk group were more likely to have been quarantined than the low-risk group due to unprotected contact with SARS patients. - 49% felt underappreciated by their co-workers, and 42% did not feel appreciated by society for the nature of their work. - 60% felt friends and neighbours had avoided them, and 36% felt their friends and family had been avoided due to concerns of contracting SARS. - Univariate logistic regression showed that taking care of only one patient with SARS was more stressful than taking care of none or two or more. Working in a high-risk unit, attending only one SARS patient, perception of personal risk, impact on work life, and depressive affect contributed to the presence of post-traumatic stress symptoms. - Multivariate linear regression showed that depressive affect was impacted by symptoms of avoidance, hyperarousal, and intrusion. Level of avoidance was positively associated with greater impact on personal life, impact on work life, and depressive affect. Level of hyperarousal was similarly influenced by the perception of one's own risk, impact on personal life, impact on work life, and depressive affect. Intrusive symptoms were associated with taking care of only one patient with SARS, perception of one's own risk, impact on work life, and depressive affect. |
| Su et al. (2007) | | |
| Design | | Cross-sectional survey |
| Participants | | 102 nurses (100% female; 70 from SARS units, 32 from non-SARS units) |
| Country | | Taiwan |
| Time period | | A 7-week period ending 30^th^ June 2003, during the peak of the outbreak. Participants completed behavioural assessments weekly or bi-weekly. |
| Incident type  Aim | | SARS outbreak  To assess the rapidly changing psychological status of nurses during the acute phase of the SARS outbreak |
| Measures | | Beck Depression Inventory; Speilberger Trait Anxiety Inventory; Chinese version of the Davidson Trauma Scale; Pittsburgh Sleep Quality Index; Sheehan’s Disability Scale; Family APGAR Index; scale developed by authors to measure changes in knowledge and understanding of SARS, perceived negative feelings toward SARS, and positive attitude toward caring for patients with SARS. |
| Key findings | | - 27.5% of nurses were symptomatic of depression but there was no significant difference between units. - Symptoms of depression decreased as the SARS epidemic decreased. Findings show that nurses working in SARS units in the beginning reported moderate levels of depression that gradually diminished to normal levels a month later, while nurses in the non-SARS units were not clinically depressed throughout the study. Anxiety demonstrated a similar pattern. - Nurses in the SARS units and those in non-SARS units with uncertainty about displacement reported higher symptoms of post-traumatic stress. SARS unit nurses reported higher post-traumatic stress symptoms across the four-week period than non SARS unit nurses. - SARS unit nurses experiences significantly greater insomnia than non SARS unit nurses but this improved over the four week period. - Knowledge of SARS improved over time in SARS unit nurses but not non-SARS unit nurses. - Positive attitudes and less negative feelings toward SARS patient care was associated with less psychiatric morbidity – continuous positive coping behaviour protected against stress. - For SARS unit nurses, significant reduction in mood ratings, insomnia rate and perceived negative feelings as well as increasing knowledge and understanding of SARS at the end of the study indicated gradual psychological adaptation. - Occurrence of psychiatric symptoms was linked to direct exposure to SARS patient care, previous mood disorder history, younger age and perceived negative feelings. Positive coping attitude and strong social and family support may protect against acute stress. |
| Tam, Pang, Lam, and Chiu (2004) | | |
| Design | | Questionnaire |
| Participants | | 652 participants from across two hospitals that were major sites of SARS outbreaks, PWH and AHNH (62% nurses, 24% healthcare assistants, 2% medical professionals). |
| Country | | Hong Kong |
| Time period | | June 2003, after the peak of the SARS outbreak but before the country had been declared SARS free. |
| Incident type  Aim | | SARS outbreak  To investigate the origin of stress and psychological morbidity among frontline healthcare workers in response to the SARS outbreak. |
| Measures | | General Health Questionnaire; scales designed to measure socio-demographics, exposure to SARS patients, subjective ratings of physical health |
| Key findings | | - 68% reported significant or severe levels of job stress, 32% reported mild or moderate stress levels. Among the high-stress level group, 79% reported low levels of job-related stress before the outbreak. Over 70% of respondents expressed the view that the most distressing aspect was HCWs becoming infected. - Variables significantly associated with high job-related stress included: younger age (below median age), being a nurse, experience of direct care for SARS patients, and poorer self-rated physical health. - 56.7% scored above the threshold on GHQ. Higher scores were associated with job-related stress, poor self-rated physical health, and be less willing to work in SARS units, in addition to being female and nursing professionals. - Significant predictors of psychological morbidity included: being female, having poor self-rated physical health, high level of job-related stress, and inadequate support (in the form of counselling and psychological support from the employer, and insurance and compensation). - Sources of stress included work, personal, and role. The high-stress group experienced significantly more of these stressors than the low-stress. - 90% of participants agreed that they now had an increased awareness of personal and environmental hygiene and valued gatherings with family members more. They also felt more devoted to helping others, as they had survived the disaster. Participants who agreed that ‘death and disease could be unexpected and near’ had a significantly higher relative risk of being in the high-stress group compared with those who disagreed. - Stress was mediated by appraisal of situations, risk perception and sense of control. - Despite the Hospital Authority of Hong Kong setting up a hotline and counseling service for distressed members of staff, the uptake was low and frontline healthcare workers seldom considered professional counseling. |
| Verma et al. (2004) | | |
| Design | | Questionnaire |
| Participants | | 1050 participants – 721 GPs (60.6% males; 10.7% has worked in a SARS affected hospital or clinic and 4.4% had been directly involved in the care of SARS patients); and 329 TCM practitioners (59% male; 1.2% reported working in a SARS-affected hospital or clinic and only 1 had direct contact with a patient with SARS). |
| Country | | Singapore |
| Time period | | Two months after the 2003 SARS outbreak |
| Incident type  Aim | | SARS outbreak  To examine the psychological impact of SARS on general practitioners’ and traditional Chinese medicine practitioners in Singapore. |
| Measures | | General Health Questionnaire; Impact of Event Scale-Revised; scales designed to measure the perception of stigma |
| Key findings | | - Significantly more GPs worked in SARS affected facilities and cared for patients with SARS than the TCM practitioners. GPs who cared for patients with SARS reported significantly higher GHQ and IES-R scores for intrusion, avoidance, hyperarousal, and stigma than GPs not involved in care of patients with SARS. GPs were significantly more likely to have a higher GHQ and IES-R scores than TCMs. - Mean score of the GHQ somatic, anxiety and social dysfunction subscales were significantly higher in GPs compared to TCM Practitioners. - GHQ total score and subscales were significantly correlated with the IES-R and stigma subscales (in Singapore the government named the clinics where SARS cases had been, which could impact income as patients may stay away for fear of infection). - Many HCWs were frightened of infecting self, family or loved ones (37.5%) and uncontrolled spread in the community (27.5%). 16% noted financial problems due to drop in patient attendance. Many HCWs said that prompt, accurate and transparent information, updated guidelines (32.6%), setting up screening stations and directing patients to the hospital predetermined for SARS treatment (15.8%), and better provision of protective gear (15.4%) would have helped. |
| Wong et al. (2004) | | |
| Design | | Cross-sectional survey |
| Participants | | 137 general practitioners (112 male, 24 female) |
| Country | | Hong Kong |
| Time period | | End of May 2003 as hospitals were beginning to return to normal functioning |
| Incident type  Aim | | SARS outbreak  To explore the impact of SARS on general practitioners in Hong Kong. |
| Measures | | Scales designed by authors to measure demographics, training for SARS, anxiety, clinical practices, and use of screening tools. |
| Key findings | | - Female doctors were significantly more worried about infecting their family and perceived their families to be more anxious about infection. Young and middle aged doctors reported their quality of life being significantly more affected than older colleagues. Exposure to SARS and working districts had no impact on anxiety levels. - Doctors in the exposed group that were more frightened of dealing with SARS reported significantly stronger worries about infecting family and impact on quality of life, compared to those in the non-exposed group. - Doctors working in high infection districts were more likely to wear gowns and close clinics due to having treated suspected cases. The majority of doctors reported that SARS had caused them to change their clinical practices, including greater use of PPE, ordering more blood tests, overprescribing antibiotics, keeping a greater distance from patients, and insisting patients wear masks during consultations. - Most doctors tried to protect family from infection by taking a shower, washing hands before going home, and disinfecting their home regularly. A small number also wore masks at home, sent their family away, or stayed away from home. - Doctors who were exposed to SARS or worked in high infection districts were less likely to quarantine themselves. |
| Wong et al. (2005) | | |
| Design | | Cross-sectional survey |
| Participants | | 466 HCWs (65.7% female, 34.3% male; 45% healthcare assistants, 37% nurses, 32% doctors) |
| Country | | Hong Kong |
| Time period | | Late June-early July 2003 as hospitals were returning to normal functioning |
| Incident type  Aim | | SARS outbreak  To examine the degree and sources of mental distress and coping strategies adopted by HCWs of emergency departments during the SARS outbreak. |
| Measures | | Chinese version of the Brief Cope Questionnaire; distress was measured using a single item designed by the authors; scale designed by the authors to measure sources of distress |
| Key findings | | - Distress was significantly higher in nurses than healthcare assistants but not doctors. There was no difference in distress across age, sex, hospitals, rank, or years of service. - Doctors were significantly more likely than nurses and healthcare workers to adopt planning coping strategies, whereas nurses were significantly more likely than doctors to use behavioral disengagement, and healthcare workers were significantly more likely that doctors to use self-distraction. - Distress was significantly correlated with six sources of distress (vulnerability/loss of control; health of self; spread of virus; health of family and others; changes in work; and being isolated). Nurses reported significantly higher distress than doctors across all six sources. |
| Wu et al. (2009) | | |
| Design | | Cross-sectional survey |
| Participants | | 549 HCWs (76.5% female, 23.5% male; 37.6% nurse, 20.7% doctor, 22.1% technician, 19.6% other) |
| Country | | China |
| Time period | | 2006 (at least 30 months after the outbreak) |
| Incident type  Aim | | SARS outbreak  To examine the psychological impact of the SARS outbreak on hospital employees in Beijing. |
| Measures | | Impact of Events Scale-Revised; scales designed by the authors to measure exposure to the SARS outbreak, other exposure to traumatic events prior to the outbreak, perceptions of SARS-related risk during the outbreak, and current fear of SARS |
| Key findings | | - 10% reported having had high levels of post-traumatic stress symptoms at some time during the three years following the outbreak. Those under 50 were significantly more likely to have experienced high PTS symptoms. Event and work exposure, being quarantined, and having a fiend or relative who contracted SARS were all associated with higher PTS symptoms. Those with higher PTS symptoms also reported higher perceived risk during the SARS outbreak and higher current levels of SARS fear. - Altruistic acceptance of risk had an independent protective effect against high PTS symptom levels. - High current PTS symptom levels three years after the outbreak was significantly associated with being single, and low household income. Outbreak exposure did not significantly predict this persistence in PTS symptoms. - Married hospital employees reported elevated fears of another SARS outbreak. Levels of perceived risk during the outbreak and PTS symptom levels were positively related to current fear levels. |
| Xing et al. (2020) | | |
| Design | | Cross-sectional survey |
| Participants | | 548 HCWs from eight provinces and cities in China |
| Country | | China |
| Time period | | At some period during the outbreak |
| Incident type  Aim | | Covid-19  To study the relationship between personality traits and mental health conditions of HCWs as a basis for implementing targeted education on mental health. |
| Measures | | Symptom Checklist-90; scale designed by authors to measure demographics, public health training, family support, health worries, level of exposure, and whether HCWs have received recent medical observation. |
| Key findings | | - HCWs reported significantly higher somatization, obsessive-compulsive anxiety, phobic anxiety, and psychoticism than the norm for the general population. However, interpersonal sensitivity was significantly lower. - Factors associated with poorer mental health included suspicion of being infected, health worried about family and self being infected, age, exposure to infection, and level of family support. |
